# Colloidal InAs Tetrapods: Impact of Surfactants on the Shape Control

**DOI:** 10.1021/jacs.3c03906

**Published:** 2023-08-08

**Authors:** Zheming Liu, Roberta Pascazio, Luca Goldoni, Daniela Maggioni, Dongxu Zhu, Yurii P. Ivanov, Giorgio Divitini, Jordi Llusar Camarelles, Houman Bahmani Jalali, Ivan Infante, Luca De Trizio, Liberato Manna

**Affiliations:** ^†^Nanochemistry, ^‡^Analytical Chemistry, ^§^Materials Characterization, ^∥^Electron Spectroscopy and Nanoscopy, ^⊥^Photonic Nanomaterials and ^#^Chemistry Facility, Istituto Italiano di Tecnologia, Via Morego 30, 16163 Genova, Italy; % Dipartimento di Chimica e Chimica Industriale, Università di Genova, 16146 Genova, Italy; & Dipartimento di Chimica, Università degli Studi di Milano, Via Golgi 19, 20133 Milano, Italy; @ BCMaterials, Basque Center for Materials, Applications, and Nanostructures, UPV/EHU Science Park, Leioa 48940, Spain; $ Ikerbasque Basque Foundation for Science Bilbao 48009, Spain

## Abstract

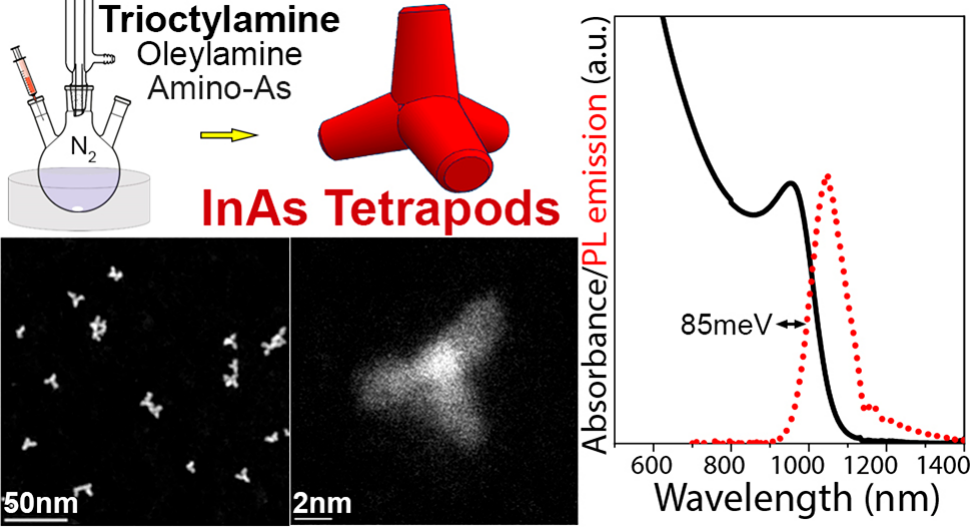

We have approached the synthesis of colloidal InAs nanocrystals (NCs) using amino-As and ligands that are different from the commonly employed oleylamine (OA). We found that carboxylic and phosphonic acids led only to oxides, whereas tri-*n*-octylphosphine, dioctylamine, or trioctylamine (TOA), when employed as the sole ligands, yielded InAs NCs with irregular sizes and a broad size distribution. Instead, various combinations of TOA and OA delivered InAs NCs with good control over the size distribution, and the TOA:OA volume ratio of 4:1 generated InAs tetrapods with arm length of 5–6 nm. Contrary to tetrapods of II–VI materials, which have a zinc-blende core and wurtzite arms, these NCs are entirely zinc-blende, with arms growing along the ⟨111⟩ directions. They feature a narrow excitonic peak at ∼950 nm in absorption and a weak photoluminescence emission at 1050 nm. Our calculations indicated that the bandgap of the InAs tetrapods is mainly governed by the size of their core and not by their arm lengths when these are longer than ∼3 nm. Nuclear magnetic resonance analyses revealed that InAs tetrapods are mostly passivated by OA with only a minor fraction of TOA. Molecular dynamics simulations showed that OA strongly binds to the (111) facets whereas TOA weakly binds to the edges and corners of the NCs and their combined use (at high TOA:OA volume ratios) promotes growth along the ⟨111⟩ directions, eventually forming tetrapods. Our work highlights the use of mixtures of ligands as a means of improving control over InAs NCs size and size distribution.

## Introduction

Near-infrared (NIR) emitting nanocrystals (NCs) are becoming increasingly attractive for exploitation in several state-of-the-art applications including solar concentrators,^1,2^ night vision,^3^ telecommunications,^4^ lasing,^5^ and bioimaging.^6,7^ From a colloidal synthesis standpoint, the most developed NIR emitting NCs incorporate toxic Pb and Hg elements.^8−10^ Consequently, synthesizing alternative NIR NC materials with superior optical properties while also adhering to the EU’s “Restriction of Hazardous Substances” (RoHS) directives is a currently pressing, open challenge.^11−16^ The most promising NCs to address this are InAs ones because their optical bandgap can be adjusted from the visible to the whole NIR range.^17−20^

The synthesis of InAs NCs, akin to that of III–V semiconductor materials, is a challenging task and is limited by the choice of suitable pnictide precursors.^21^ Currently, the most widely used As precursors for this purpose include As[M(CH_3_)_3_]_3_ (M = Si or Ge; TMM-As).^18,22−32^ Although the synthesis routes based on TMM-As have reached maturity, the inherent toxicity, high cost, and limited commercial availability of such precursor are critical shortcomings for a viable synthesis of InAs NCs. In recent years there has been a rapid development of synthesis strategies that utilize alternative As precursors, with the most promising being As[N(CH_3_)_2_]_3_ (amino-As), which is less toxic, less expensive, and more commercially available than TMM-As.^32−38^ The use of amino-As for the synthesis of InAs NCs requires a reducing agent to promote As^3+^ → As^3–^ reduction. The strength of this reducing agent plays a significant role in governing the kinetics of nucleation and growth of the NCs.^32−38^ Currently, the best reducing agent available is alane *N*,*N*-dimethylethylamine (DMEA-AlH_3_),^36^ which however leads to NCs with poorer optical characteristics than those achieved with TMM-As.^30,34,36,38,39^ Typically, the size distribution of InAs NCs is expressed in terms of the width of their first exciton absorption peak (measured as half-width at half-maximum, HWHM), which can be as narrow as 40 meV when employing TMM-As, while the use of amino-As yields NCs with higher values, typically above 100 meV.^30,34,36,38,39^

**Scheme 1:**
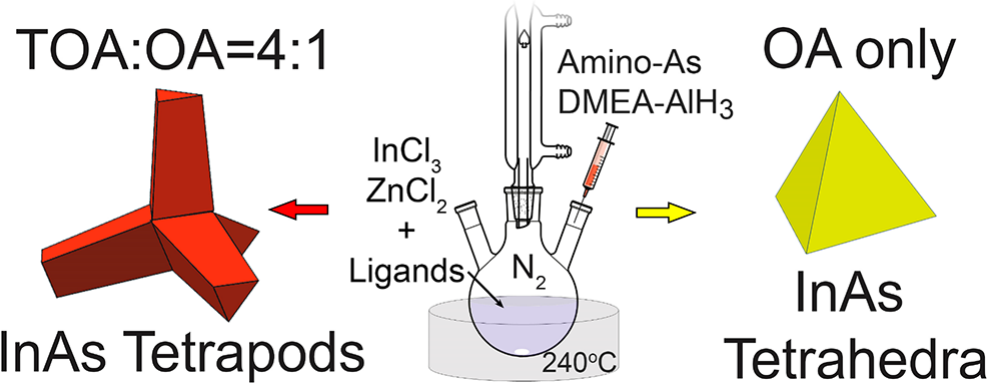
Synthesis of InAs NCs with Different Combinations of Surfactants

Additional parameters that affect the synthesis kinetics and require further investigation include the type of ligands employed.^35^ The synthesis of amino-As-based InAs NCs currently relies solely on the use of oleylamine (OA), and we will refer to NCs prepared in this way as “standard” InAs NCs.^32,35,36,38^ The motivation of the present work is that, to date, a comprehensive exploration of alternative surfactants and their impact on the nucleation and growth of InAs NCs has not yet been performed. To close this gap, here we started from a synthesis protocol developed by us in a previous work, which relies on amino-As, InCl_3_, ZnCl_2_, and DMEA-AlH_3_,^38^ and analyzed the effects of different surfactants on the structural and optical properties of the resulting InAs NCs. Specifically, we tested oleic acid, octadecylphosphonic acid, tri-*n*-octylphosphine (TOP), dioctylamine (DOA), trioctylamine (TOA), and mixtures of these ligands with OA (Scheme 1). Our findings are the following: (i) carboxylic and phosphonic acids result in the formation of In_2_O_3_ and As_2_O_3_ as a consequence of their reaction (i.e., condensation) with the amino groups present in the amino-As precursor. (ii) TOP, DOA, and TOA, when used as the sole surfactants, lead to InAs NCs with poor control over their size and shape. (iii) The combination of TOP or DOA with OA does not significantly improve the NCs size distribution. (iv) A mixture of TOA with OA leads to InAs NCs with better control over size and size distribution. Additionally, at a TOA:OA volume ratio of 4:1 InAs tetrapods are formed, which is a remarkable result as such shape is very little known for this material.

Our InAs tetrapods are monocrystalline, with core size of ∼2.5 nm and arm length of 5–6 nm, and have the cubic zinc-blende crystal structure with arms growing along the ⟨111⟩ directions. They have a narrow absorption exciton peak at ∼950 nm, with HWHM values as low as 85 meV (among the lowest reported for InAs NCs) and a photoluminesce (PL) emission at 1050 nm. The HWHM values are quite low if one considers that InAs NCs of these sizes should be in the strong quantum confinement regime where even small variations in size should result in a very broad absorption peak. Our calculations indicate that the bandgap energy of InAs tetrapods is not affected by the length of the arms when they are longer than ∼3 nm. Hence, even with a poor arm length distribution, the HWHM of InAs tetrapods is narrow if the core size is kept relatively constant.

Nuclear magnetic resonance (NMR) indicated that the surface of the tetrapods was mostly passivated by OA, with only a small fraction of weakly bound TOA. These findings were rationalized through classical molecular dynamics (MD) simulations under conditions mimicking the experiments, which revealed that TOA, given its steric hindrance, binds weakly to the edges and corners of InAs NCs, while OA tends to passivate the (111) facets. When employing more TOA than OA, the surface covered by OA decreases, and this should allow the NCs to grow along the ⟨111⟩ directions, promoting the formation of tetrapods.

Overall, our approach, combining experiments and modeling, suggests that the use of TOA in conjunction with OA enables fast monomer access to the surface of InAs NCs, and this combination of ligands can be exploited to produce larger NCs. Additionally, a specific ratio of TOA and OA can alter the reaction kinetics to the point that it promotes the formation of tetrapod-shaped NCs.

## Experimental Section

### Materials

Indium(III) chloride (InCl_3_, 99.999%, Sigma-Aldrich), zinc(II) chloride (ZnCl_2_, 99.999%, Sigma-Aldrich), tris(dimethylamino)arsine (amino-As, 99%, Strem), alane *N*,*N*-dimethylethylamine complex solution (DMEA-AlH_3_, 0.5 M solution in toluene, Sigma-Aldrich), triethyloxonium tetrafluoroborate (Et_3_OBF_4_, 97%, Sigma-Aldrich), oleylamine (OA, 98%, Sigma-Aldrich), trioctylamine (TOA, 98%, Sigma-Aldrich), dioctylamine (DOA, 97%, Sigma-Aldrich), tri-*n*-octylphosphine (TOP, 97%, Strem), oleic acid (90%, Sigma-Aldrich), octadecylphosphonic acid (>99%, PCI synthesis), toluene (anhydrous, 99.8%, Sigma-Aldrich), ethanol (anhydrous, 99.8%, Sigma-Aldrich), toluene-*d*_8_ (anhydrous, 99.6%, Sigma-Aldrich), ethyl acetate (anhydrous, 99.8%, Sigma-Aldrich), hexane (anhydrous, 95%, Sigma-Aldrich), and *N*,*N*-dimethylformamide (DMF, anhydrous, 99.8%, Sigma-Aldrich) were used without further purification.

### Preparation of the As Precursor

The As precursor was prepared following a previously reported method by Srivastava et al.^36,37^ In a N_2_-filled glovebox, 0.2 mmol of amino-As was dissolved in 0.5 mL of degassed oleylamine at 40 °C for 5 min until no bubbles further evolved.

### Synthesis of InAs Nanocrystals

In a typical synthesis, 0.2 mmol of InCl_3_, 1 mmol of ZnCl_2_, and 5 mL of the desired ligand (or combination of ligands; see below) were loaded into a 100 mL three-necked flask under an inert atmosphere and dried at 120 °C under vacuum for 1.5 h. The mixture was heated to 240 °C, and then the As precursor was rapidly injected into the flask, quickly followed by the injection of 1.2 mL of the DMEA–AlH_3_ toluene solution. The reaction was then monitored over time (Figures S1–S4 of the Supporting Information) and was then quenched by removing the flask from the heating mantle and allowing it to cool down. When the reaction mixture reached a temperature of 90 °C, the flask was transferred into a N_2_-filled glovebox. The NCs were purified by the addition of toluene and ethanol and precipitated by centrifugation at 4000 rpm for 5 min. The precipitate was dispersed in toluene and centrifuged at 4000 rpm for 5 min to remove byproducts. The supernatant was collected for further characterizations. All the purification steps were performed under a N_2_ atmosphere. This synthesis scheme was adopted by employing either (i) only one type of ligand among OA, DOA, TOA, TOP, oleic acid, and octadecylphosphonic acid (5 mL of the desired ligand in each case) or (ii) combinations of TOP+OA, DOA+OA, and TOA+OA (with volume ratios ranging from 1:4 to 4:1, having a total volume of 5 mL).

### Optimization of the InAs Tetrapods Synthesis

In order to optimize the synthesis of InAs tetrapods, we employed 0.2 mmol of InCl_3_, 1 mmol of ZnCl_2_, and 5 mL of a TOA:OA mixture (volume ratio 4:1) which were loaded into a 100 mL three-necked flask under an inert atmosphere and dried at 120 °C under vacuum for 1.5 h. The mixture was heated to the desired injection temperature (see below). The As precursor was then injected into the flask, quickly followed by the injection of 1.2 mL of a DMEA–AlH_3_ toluene solution. The reaction was performed at the desired temperature (see below) for 6 h. The NCs growth was stopped by removing the flask from the heating mantle and allowing it to cool down. When the reaction mixture reached a temperature of 90 °C, the flask was transferred into a N_2_-filled glovebox. The NCs were washed by the addition of toluene and ethanol and precipitated by centrifugation at 4000 rpm for 5 min. The precipitate was dispersed in toluene and centrifuged at 4000 rpm for 5 min to remove byproducts. The supernatant was collected for further characterizations. All of the purification steps were performed under a N_2_ atmosphere. The optimization of the synthesis occurred by systematically varying (1) the injection temperature from 160 to 240 °C and (2) the reaction temperature from 240 to 300 °C.

### Ligand Stripping Procedure

A ligand stripping procedure was employed to prepare NC samples for high-resolution transmission electron microscopy (to avoid the contamination from organic ligands), and it was performed by following the procedure reported by Rosen et al.^40^ In a N_2_-filled glovebox, 0.5 mL of a NC dispersion was added to 1 mL of hexane in a glass vial, and then 1 mL of a solution of Et_3_OBF_4_ in DMF (100 mM) was added into the vial. After shaking the vial for several seconds, the NCs were transferred from the hexane into the DMF phase. The NCs dispersed in DMF were precipitated by the addition of toluene followed by centrifugation at 4000 rpm for 5 min. To remove residual organic ligands, the washing procedure was repeated twice and the resulting NCs were dispersed in DMF.

### X-ray Diffraction (XRD)

XRD patterns were recorded on a Malvern-PANalytical third-generation Empyrean X-ray diffractometer, equipped with a 1.8 kW Cu Kα ceramic X-ray tube, a polycapillary X-ray lens, and a GaliPIX3D solid state area detector, operating at 40 kV and 45 mA in 2D mode. Concentrated NC solutions were drop-cast on a zero-diffraction quartz substrate in the glovebox and then collected under ambient conditions at room temperature. XRD data were analyzed by the HighScore Plus 5.1 software from PANalytical.

### Transmission Electron Microscopy (TEM)

Diluted NC solutions were drop-cast onto copper TEM grids with an ultrathin carbon film. Low-resolution TEM images were acquired on a JEOL JEM-1400Plus microscope with a thermionic gun (W filament) operated at an acceleration voltage of 120 kV. High-resolution scanning transmission electron microscopy (HRSTEM) images were acquired on a probe-corrected ThermoFisher Spectra 300 STEM operated at 300 kV. Images were acquired on a high-angle annular dark field (HAADF) detector with a current of 30 pA. Compositional maps were acquired using Velox, with a probe current of ∼200 pA and rapid rastered scanning. The energy-dispersive X-ray (EDX) signal was acquired on a Dual-X system comprising two detectors on either side of the sample, for a total acquisition angle of 1.76 Sr. HAADF HRSTEM tilt series were acquired by tilting the double tilt sample holder at −35°, 0°, and 35°; the axis of rotation is parallel to the horizontal line of the images.

### Optical Measurements

The absorption spectra were recorded with a Varian Cary 5000 UV–vis–NIR spectrophotometer. The samples were prepared with 1 cm path length quartz cuvettes with airtight screw caps under an inert glovebox. The steady-state PL and time-resolved PL measurements were performed on a Edinburgh FLS900 fluorescence spectrometer equipped with an Xe lamp and a monochromator for steady-state PL excitation and a time-correlated single photon counting unit coupled with a Edinburgh Instruments EPL-510 pulsed laser diode (λ_ex_ = 508.2 nm, pulse width = 177.0 ps) for time-resolved PL. The PLQY measurements were performed using the Edinburgh FLS900 fluorescence spectrometer equipped with an integrating sphere excited at 800 nm using the output of continuous xenon lamp. All NC solutions were diluted to an optical density of around 0.15 at 800 nm.

### Nuclear Magnetic Resonance (NMR)

Samples for NMR analyses were dissolved in 0.5 mL of toluene-*d*_8_ and transferred to 5 mm disposable tubes (Bruker). The ^1^H and nuclear Overhauser effect spectroscopy (2D ^1^H–^1^H NOESY) NMR experiments were acquired at 298 K by using a Bruker Avance III 600 MHz (600.13 MHz) spectrometer, equipped with a 5 mm QCI cryoprobe with *z* shielded pulsed-field gradient coil. Before acquisitions, the automatic matching and tuning were run, the homogeneity was optimized, and the 90° angle was adjusted on each sample tube^41^ by using Bruker’s automatic routines.

^1^H NMR spectra were recorded by accumulating 128 transients, without dummy scans, with 65536 digit points and an interpulse delay of 30 s, over a spectral width of 19.84 ppm, centered at 6.175 ppm. The acquisition time was 2.75 s. An exponential apodization function of 0.3 Hz was applied to FIDs before the Fourier transform was applied. Spectra were manually adjusted in the phase and automatically baseline corrected. 2D ^1^H–^1^H NOESY experiments were run by using the pulse sequence noesygppphpp from Bruker’s library.^42,43^ After 32 dummy scans, 16 transients were accumulated, with 2048 digit points and 256 increments, a mixing time of 300 and 100 ms, respectively, and a relaxation delay of 2 s, over a spectral width of 9.8 ppm with the offset positioned at 4.9 ppm.

Diffusion NMR studies were performed on a Bruker DRX400 spectrometer (400.13 MHz), equipped with a Bruker 5 mm BBI Z-gradient probe head, affording a maximum gradient strength of 53.5 G/cm. All of the spectra were acquired at 300 K. ^1^H diffusion order spectroscopy (DOSY) experiments were acquired using a ledbp pulse sequence (ledbpgp2s of the Bruker library),^44^ using a diffusion time (Δ) of 150–200 ms and a total gradient pulse duration (δ) of 3–4 ms. The gradient strength (*G*) was incremented in 32 steps from 5 to 95% of its maximum value following a function with a SINE.100 shape.

The following equation describes the intensity decay as a function of the power of the applied gradient strength *G*^2^:
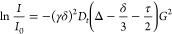
in which *I* is the observed intensity, *I*_0_ is the nonattenuated signal intensity, *D*_*t*_ is the diffusion translational coefficient, γ is the ^1^H gyromagnetic ratio, and τ is the time between bipolar gradients. We obtained diffusion coefficients by analyzing the signal intensity decay as a function of the gradient strength *G* of at least three different resonances by using a least-squares linear fitting, from the slope of which *D*_*t*_ can be calculated.

### k·p Calculations

We used single-band k·p Hamiltonians to describe noninteracting electron and heavy hole energy band-edge states of InAs NCs with spherical and tetrahedral symmetry. The latter symmetry is valid for describing both the quantum dot (with arm length ∼0) and the tetrapod (with arm length >0). We considered the interaction between the conduction band minimum and the valence band maximum using the Kane parameter, and we also incorporated the effect of spin–orbit coupling in the k·p Hamiltonians. To solve the Schrödinger equations of the 1-band electron and hole, we used the finite element method provided by the COMSOL 6.1 software. More details on the choice of the parameters used for modeling are provided in the Supporting Information.

### Classical Molecular Dynamics (MD)

To prepare the InAs NC models, we started with an InAs zinc-blende bulk structure (*F*43*m* space group). The bulk structure was cleaved along the In-rich (111) facets to yield a regular tetrahedron with an edge length of 4.5 nm. We then removed the vertex tips to expose a small fraction of the As-rich (111) facets. The resulting InAs NC model was found to be non-neutral with an In:As ratio of 1.15 (exact stoichiometry In_889_As_776_), which was consistent with experimental observations. The height of the InAs core in the NC was measured to be 4.0 nm. To balance the charge of the starting InAs NC, we introduced 339 Cl ions, whose position on the NC surface was determined using classical molecular dynamics (MD) simulations in the canonical (NVT) ensemble, at constant number of particles *N*, temperature *T* (300 K), and fixed volume *V*. Initially, the Cl ions were unbound and randomly placed inside a cubic simulation box with a side length of 10 nm. We restrained the positions of the InAs core atoms around their starting positions during the initial stages of the simulations to ensure gradual equilibration of the system, until Cl ions were bound to the surface. The simulations were conducted for 100 ps using an integration time step of 0.5 fs. To improve the sampling of our MD simulations, we prepared various Cl-passivated InAs NC models starting from random unbound Cl positions.

For the calculations that include the OA:TOA mixture, we initially performed NVT MD simulations to equilibrate the systems employing toluene as a solvent, followed by a simulation for 50 ns using an isothermal–isobaric environment (NpT) with a constant pressure of 1 atm and temperature of 300 K. Toluene was initially used to facilitate the equilibration of the OA and TOA ligands toward the NC surface and also to prevent the formation of artificial vacuum bubbles during the equilibration. After the equilibration step, toluene was removed from the simulation boxes, as it is known to evaporate during the hot-injection synthesis. This left OA and TOA acting simultaneously as both ligands and solvent. For all the OA:TOA ratios (see Table S1), an equilibration run of 1 ns at NVT conditions was followed by a production NpT run for 100 ns with an integration time step of 1 fs.

The simulations performed in this study, along with all others, were conducted using the GROMACS 2021.3 package.^45^ The MD simulations utilized a smooth particle mesh Euler method (SPME) with beta-Euler splines,^46^ as well as a 1 nm short-range cutoff, to compute both the LJ and the Coulombic terms. The spacing between consecutive grid points was set to 1 Å. The force-field parameters for the InAs core and the bound Cl ions were previously fitted by some of us in a separate study using a combination of Lennard-Jones and Coulombic terms. The OA and TOA FF parameters were taken using MATCH,^47^ a toolset of program libraries aimed at the assignment of atom types and force field parameters in organic molecules by comparison against a data set of chemical fragments. All FF parameters are given in Table S2.

## Results and Discussion

At first, we aimed to explore which ligands, other than OA, could be used to synthesize InAs NCs via the amino-As route. To this end, we followed our recently reported synthesis protocol^38^ and substituted OA with various ligands, including oleic acid, octadecylphosphonic acid, TOP, DOA, or TOA (see the Experimental Section for details). The use of either oleic acid or octadecylphosphonic acid led to bulk-like In_2_O_3_ and As_2_O_3_ precipitates, possibly due to the condensation of the acids with the amines present in the amino-As precursor (Figure S5). In contrast, TOP, DOA, and TOA resulted in the formation of InAs NCs with the expected zinc-blende crystal structure, as confirmed by TEM and XRD (Figure S6), yet with poor control over the size and size distribution (Figure S7).

**Figure 1:**
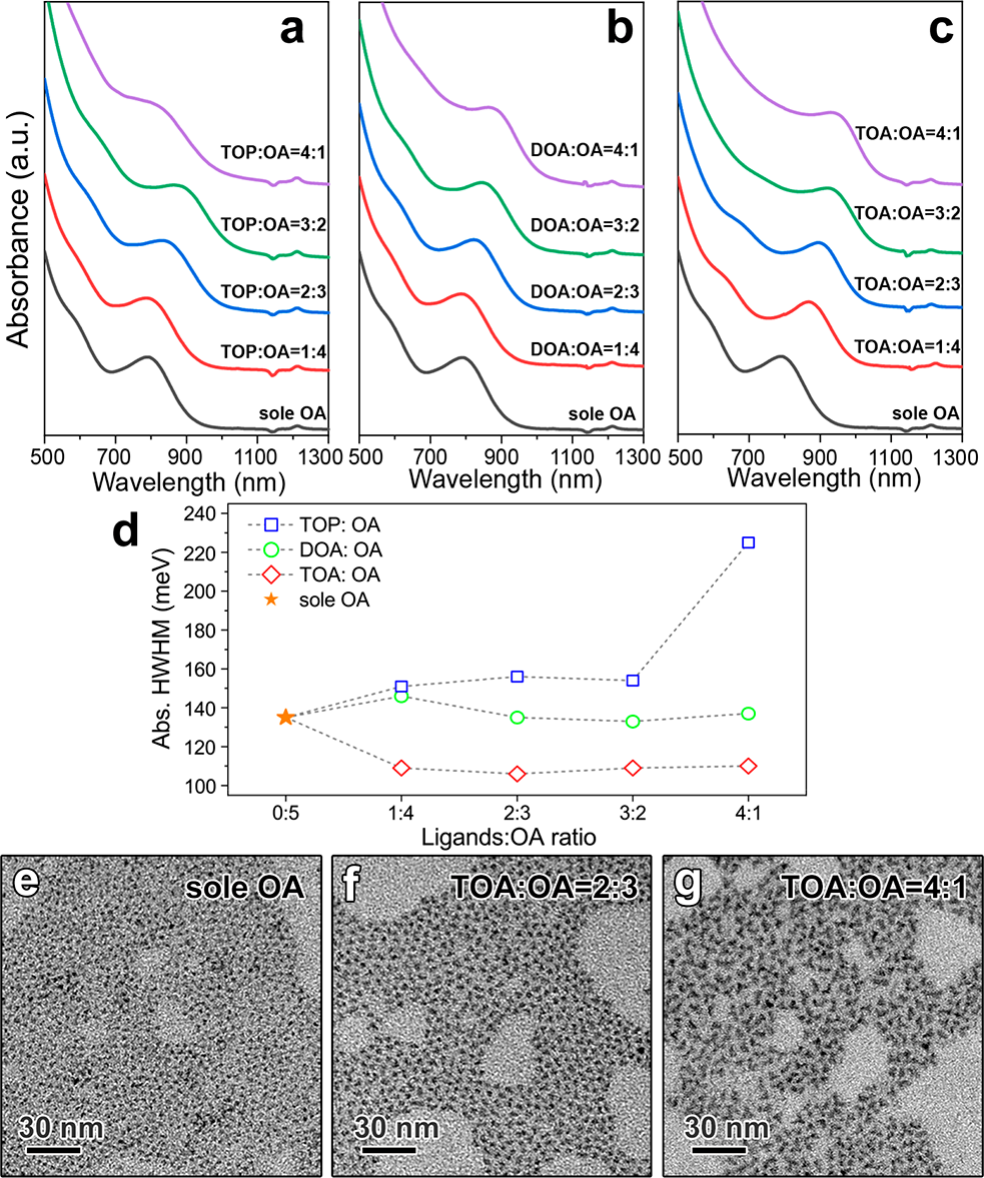
Absorption spectra of InAs NCs made with different combinations of ligands: (a) TOP+OA (reaction time 1 h), (b) DOA+OA (reaction time 2 h), (c) TOA+OA (reaction time 6 h), and (d) HWHM values of the corresponding exciton absorption peaks. TEM images of InAs NCs made with (e) only OA or with TOA:OA volume ratios of (f) 2:3 and (g) 4:1.

Since TOP, DOA, and TOA led to the formation of InAs NCs, these ligands were also tested in combination with OA under the same synthesis scheme. As a reference, we also prepared “standard” InAs NCs (that is, using solely OA) which had an absorption peak at ∼790 nm with an HWHM of ∼135 meV after a reaction time of 1 h (Table 1 and Figure S1). The combined use of TOP+OA or DOA+OA did not result in significant variations in terms of NCs size and size distribution compared with the sole OA (Figures 1, S2, and S3). Specifically, these ligand combinations delivered slightly larger NCs (absorption peak located in the 780–860 nm range) with a broader size distribution (HWHM up to 225 meV) (Table 1, Figures 1a,b,d, S2, and S3). In contrast, TOA+OA led to larger NCs (absorption peak ranging from 865 to 930 nm), with an improved size distribution (HWHM as low as ∼105 meV) (Table 1, Figures 1c,d and S4). Notably, TOA+OA led to InAs NCs growing in the size focusing regime for 6 h (i.e., the NCs grew larger, with the size distribution becoming narrower, Figure S4), as opposed to all the other cases in which the size distribution started worsening already after 1–2 h (Figures S1–S3).

**Table 1:** Optical Properties (Exciton Absorption Peak Position and HWHM) of the Samples Prepared with Different Ligands Volume Ratios

	ligands volume ratios (total volume 5 mL in each synthesis)									
	0:5		1:4		2:3		3:2		4:1	
ligand	abs (nm)	HWHM (meV)	abs (nm)	HWHM (meV)	abs (nm)	HWHM (meV)	abs (nm)	HWHM (meV)	abs (nm)	HWHM (meV)
TOP:OA	789	135	789	151	829	156	863	154	780	225
DOA:OA			788	146	822	135	845	133	863	137
TOA:OA			867	109	892	106	920	109	929	110

**Figure 2:**
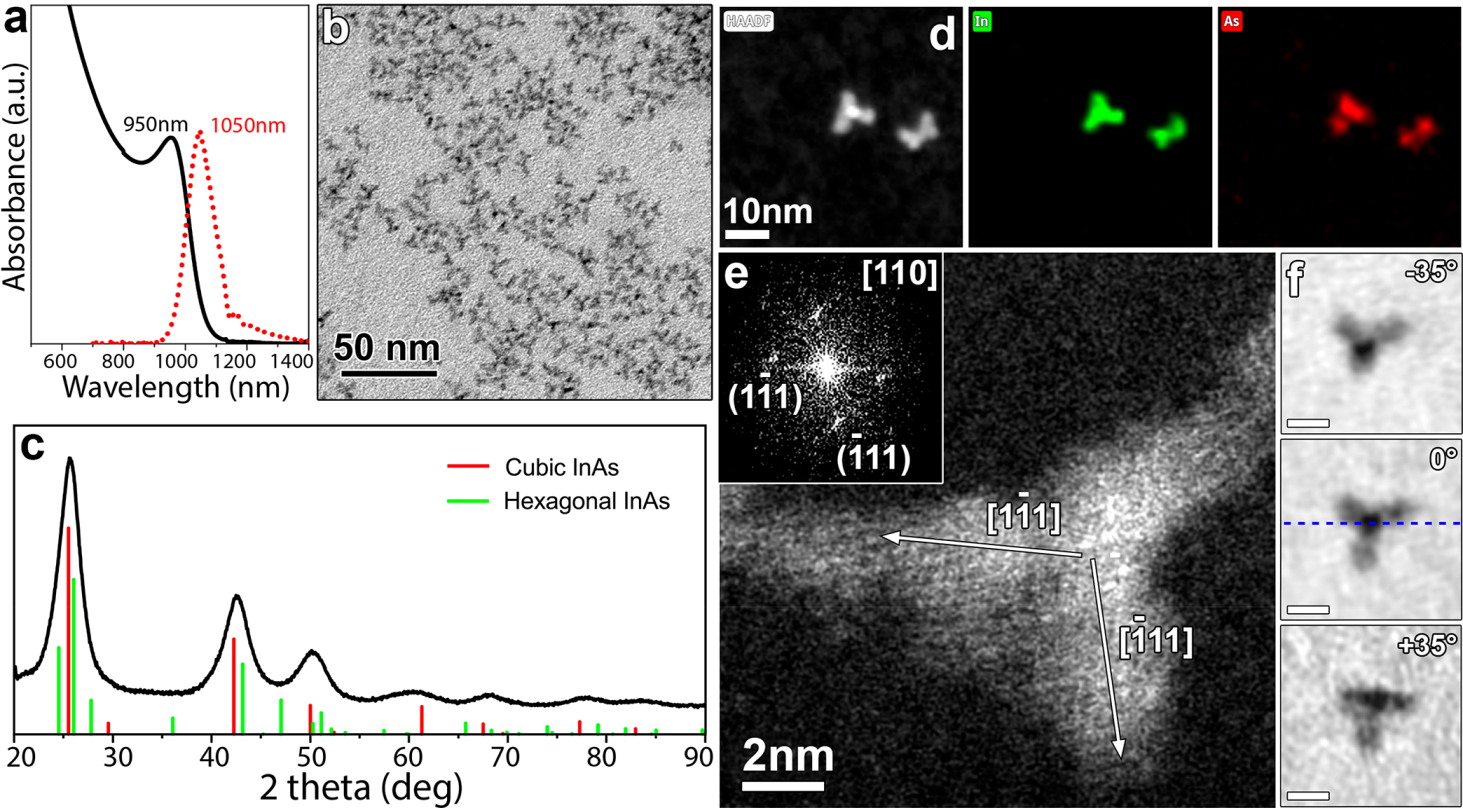
(a) Absorption and PL spectra, (b) TEM micrograph, and (c) XRD pattern of tetrapod-shaped InAs NCs. The corresponding reflections of cubic InAs (ICSD number 24518) and hexagonal InAs (ICSD number 190427) are also reported by means of red and green vertical bars, respectively. (d) HRSTEM-EDX elemental maps of two isolated tetrapods. (e) HAADF HRSTEM image of a single tetrapod aligned along the [110] zone axis, with the corresponding fast Fourier transform (FFT) (inset): the arrows indicate the crystallographic directions parallel to the arm’s growth. (f) TEM images of a single tetrapod tilted to different angles around the rotation axis indicated by dashed blue line (scale bars are 5 nm).

The interesting optical properties of the InAs NCs prepared with TOA+OA led us to investigate their morphology by TEM. The addition of TOA to OA did not fundamentally change the growth mechanism for ratios up to TOA:OA = 2:3, where a typical tetrahedral shape (Figures 1e,f and S8) is observed, as commonly seen when employing OA alone.^38,48^ Higher TOA:OA volume ratios (such as 3:2 and 4:1) delivered NCs with a branched shape (Figures 1g and S8). The unusual morphology of the NCs obtained with a TOA:OA volume ratio of 4:1 motivated us to further investigate the synthesis scheme using such a ratio. We systematically varied both the temperature at which the As precursor is injected (from 160 to 240 °C) and the reaction temperature (from 240 to 300 °C) (see Figures S9 and S10). Injecting the As precursor at moderate temperatures (200 °C) followed by NCs growth at 240 °C (reaction time of 6 h) led to NCs with a sharp exciton absorption peak at ∼950 nm and a HWHM as low as 85 meV, which is among the narrowest size distributions reported so far for amino-As-based InAs NCs (Table 1 and Figure 2a).^34^ The NCs featured a weak PL peak (PLQY ∼ 1%) at 1050 nm with an average PL lifetime of 9.9 ns (Figures 2a and S11). Such a sample was composed of NCs with a tetrapod shape (Figure 2b), which, according to XRD, had a cubic zinc-blende InAs structure (ICSD number 24518) with no XRD peaks ascribable to the InAs hexagonal phase (ICSD number 190427) (Figure 2c). The TEM analysis of the aliquots taken at different reaction time intervals revealed that (i) small (size <3 nm) tetrahedral-shaped NCs form within the first 30 s, (ii) after 1 min part of these NCs evolve into tetrapods with an average size of 3.5–4.5 nm, and (iii) after 3 min the sample is mostly composed of tetrapod-shaped NCs (Figure S12). Our findings are similar to those reported by Kim et al., who obtained tetrapods of InP, also a III–V semiconductor, featuring both core and arms in the zinc-blende phase.^49,50^

High-angle annular dark field (HAADF) scanning transmission electron microscopy (STEM) coupled with energy-dispersive X-ray (EDX) analysis confirmed that the tetrapods are made of InAs, with a core size of ∼2.5 nm and arm lengths ranging from 5 to 6 nm (Figures 2d and S13). The HRSTEM analysis of individual InAs tetrapods revealed that they are monocrystalline with the arms growing along the ⟨111⟩ crystallographic axes and exposing facets parallel to the (110) crystallographic planes (Figure 2e). The 3D structure of the InAs tetrapods was confirmed by TEM observations at various tilts: tilting the TEM grid from −35° to +35° revealed four clearly visible arms, including the central one of a tetrapod, and the projection of the central arm changed the most during the tilt series (Figure 2f).

The present InAs tetrapods feature a surprisingly narrow exciton absorption peak if one bears in mind that their size is much smaller than that of the exciton Bohr radius of InAs (∼30–40 nm).^19^ Under this regime (i.e., strong quantum confinement) one would expect huge changes in absorption peak position upon small variations of NCs size. Indeed, large variations in bandgap energies have been observed in previous works when going from 2.8 nm InAs NCs (absorption peak at 780 nm)^38^ to 9 nm ones (absorption at 1600 nm).^39^ These observations are in contrast with (i) the narrow absorption peak of InAs tetrapods, which instead do not feature a very narrow size distribution (arm lengths ranging from 5 to 6 nm), and (ii) the absorption peak position at “high energy” (∼950 nm) of InAs tetrapods despite their overall large size.

**Figure 3:**
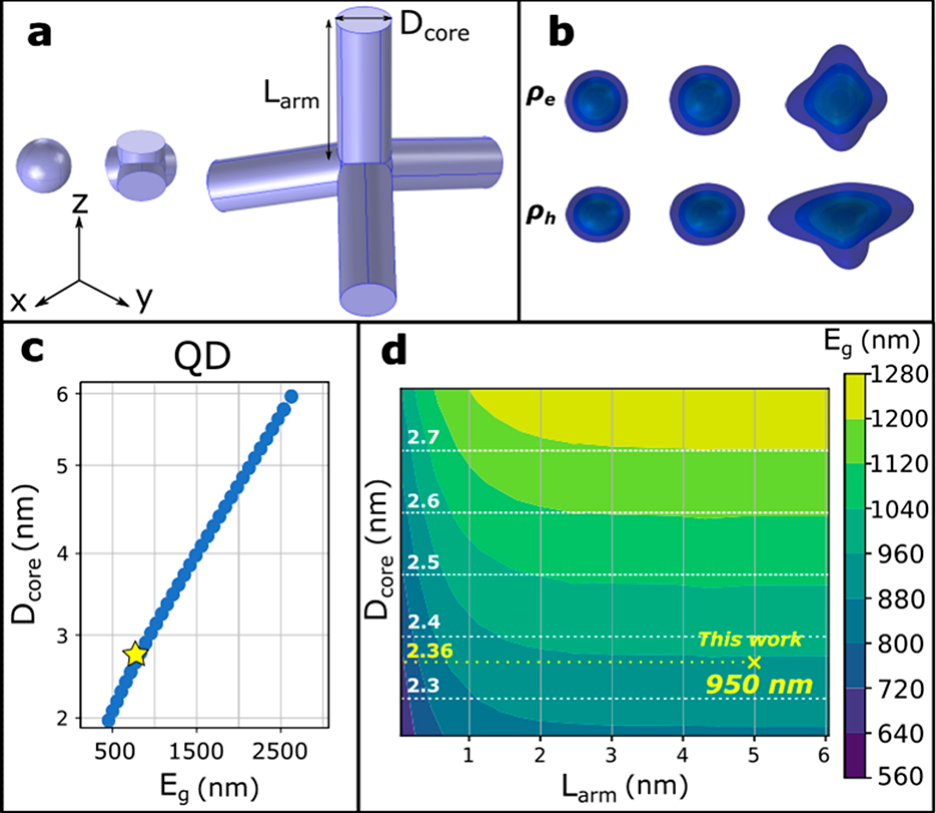
(a) Schematic of the InAs NCs shapes used in the k·p calculations: spherical, tetrahedral (with arm length (*L*_arm_) equal to 0), and tetrapod (with *L*_arm_ ≠ 0) shapes. (b) Electron (ρ_e_) (top) and hole (ρ_h_) (bottom) charge densities corresponding to InAs shapes defined in panel a. (c) Calculated bandgap energies of spherical InAs NCs as a function of the core diameter. Blue dots stand for 1-band k·p calculations, and the yellow star represents the experimental value observed in the work of Zhu et al. (∼780 nm).^38^ (d) Contour plot of the calculated bandgap energies as a function of the arm length *L*_arm_. The yellow cross shows the bandgap energy observed experimentally in this work, and the dotted lines provide an indication of the tetrapod core size at *L*_arm_ ∼ 0.

To rationalize these apparent contradictions, we performed k·p calculations on both spherical- and tetrahedral-shaped InAs tetrapods with symmetric arms of varying lengths (Figure 3a). Spherical InAs models evidenced, in agreement with the experimental data, that large variations in bandgap energy are expected even for small variations of the NCs diameter: the bandgap energy for the NC with diameter of 2.8 nm fits well with the one experimentally observed in our previous work,^38^ and subtle modifications of the NC diameter can lead to a relatively large bandgap red-shift (Figure 3c). Instead, on InAs tetrapods we found that (i) the charge density of both the band-edge electron and hole stays at the center of the tetrapods (Figure 3b) with a slight leakage of both electrons and holes into the arms; (ii) the bandgap energy red-shifts by up to 0.513 eV when moving from the pure tetrahedron (arms length equals to zero) to a tetrapod with 6 nm arm length (Figure 3d). Such a red-shift reaches saturation at around 3 nm of arm length for practically all the diameters of the tetrapod cores considered (Figure 3d). Overall, our k·p model indicated that (i) an absorption peak at 950 nm is compatible with InAs tetrapods having arm length of 5–6 nm and core size of 2.3–2.4 nm, in agreement with our HRSTEM analysis, and (ii) the narrow absorption peak experimentally observed for InAs tetrapods can be attributed to the fact that their bandgap energy remains insensitive to arm lengths when they are greater than 3 nm.

**Figure 4:**
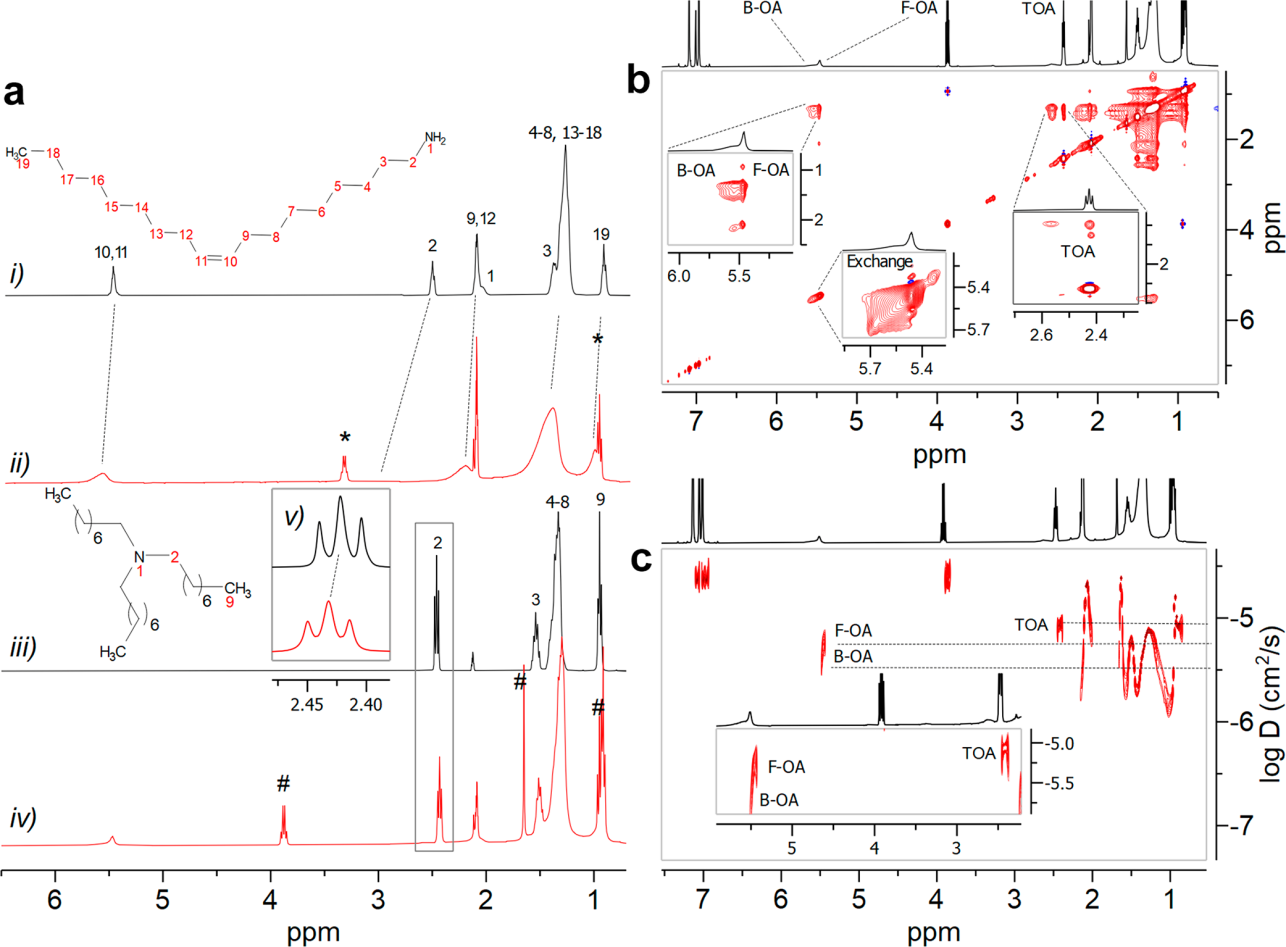
(a) ^1^H NMR spectra in toluene-*d* of (i) OA (the attribution of ^1^H OA signals has been also confirmed by a ^1^H–^13^C HSQC spectrum, Figure S21), (ii) “standard” tetrahedral InAs NCs, (iii) TOA (the attribution of ^1^H TOA signals has been also confirmed by a ^1^H–^13^C HSQC spectrum, Figure S22), (iv) InAs tetrapods, and (v) zoom-in of the CH_2_(2) of TOA (∗ = EtOH; # = ethyl acetate). (b) NOESY experiment of tetrapod InAs NCs in toluene-*d*_8_; insets are the zoom-in of the double-bond region from 5.42 to 5.66 ppm, of OA cross-peaks, and diagonal peaks (exchange) and CH_2_ of TOA at 2.43 ppm. (c) DOSY experiment with inset that reports the zoom-in of the OA and TOA diagnostic diffusion signal.

To shed light on the formation of InAs tetrapods, we first investigated their surface passivation via ^1^H NMR analysis. We also characterized, for a comparison, “standard” InAs NCs, made with sole OA. Initially, we studied the interaction of the ligands with the NCs surface via ^1^H NMR spectroscopy and then complemented our analysis with NOESY and DOSY spectroscopies. “Standard” InAs NCs made with sole OA exhibited one set of proton signals that were broadened and shifted with respect to those of free OA in the same solvent (Figure 4a, i and ii). This result suggested that OA was interacting with the NCs surface (i.e., OA molecules assume the slow tumbling regime of the NCs). ^1^H–^1^H NOESY confirmed such an interaction by yielding OA NOE cross-peaks which were positive in the case of pristine OA (Figure S15) and negative for “standard” InAs NCs (Figure S16). Indeed, the NOE sign of a ligand is dependent on its rotational correlation time (τ_c_): when a molecule is either tightly bound or temporarily interacting with the NC surface (through an in-and-out exchange process),^51^ it assumes the slow tumbling regime of the NCs and its τ_c_ value elongates, causing the sign of NOE to switch from positive (blue, for free ligands) to negative (red, for ligands interacting with the NCs). To better identify the type of interaction between OA and the surface of “standard” NCs, we employed DOSY. The 2D map (Figure S17) revealed a translational diffusion coefficient (*D*_*t*_) for OA equal to 139 μm^2^/s, which was close to the calculated value (through the Stokes–Einstein equation) of 137 μm^2^/s for NCs with a core size of 2.8 nm and an OA shell thickness of 2 nm.^52^ Such values indicated that 99.8% of OA was bound to the surface of the NCs (see the Supporting Information).^53,54^ Therefore, the peak broadening (Figure 4a, ii) and the negative NOE cross-peaks observed for OA (Figure S16) were attributed to tight binding of OA with the surface of “standard” NCs, rather than to a weak and temporary interaction.

We then acquired the ^1^H NMR spectrum of InAs tetrapods and observed two distinct OA olephynic signals (even though partially overlapped, Figure S18): one at 5.51 ppm, which was slightly broadened and shifted at lower fields compared to pristine OA, and another at 5.55 ppm, which appeared to be much more broadened and shifted. These ^1^H spectral features, together with the evidence of an exchange cross-peak in the ^1^H–^1^H NOESY 2D map (vide infra, inset “exchange” in Figure 4b), suggested a slow exchange process occurring between free (F-OA) and bound (B-OA) OA. Indeed, it must be remembered that NOESY experiments can reveal not only the cross-peaks due to NOE but also those caused by an active exchange process, occurring among couples of resonances, that leads to the transfer of the spin polarization from bound to free species, and vice versa.^55^ Additionally, the diagnostic peak of TOA, namely, the CH_2_ resonance in the position α to the nitrogen at 2.43 ppm, was slightly shifted compared to the corresponding signal of the free ligand at 2.42 ppm, and its line shape moderately broadened (full width at half-intensity of 16.2 Hz for the NCs compared to 15.9 Hz for the pristine TOA; see the inset of Figure 4a). To gain insights into the possible dynamics of the ligands at the surface of InAs tetrapods, we first analyzed the 2D ^1^H–^1^H NOESY spectrum of InAs tetrapods in solution, which showed negative (red) NOE cross-peaks, typical of species with a slow tumbling regime in solution, for B-OA, F-OA, and TOA (Figure 4b), even with a shortened mixing time (from 300 to 100 ms, aimed at minimizing the possible spin diffusion, Figure S19), suggesting that TOA interacts with the surface of InAs tetrapods.^54^ In contrast, the 2D ^1^H–^1^H NOESY spectrum on pristine TOA showed positive (blue) NOE cross-peaks (Figure S20).

The DOSY 2D map showed three distinct *D*_*t*_ values for the B-OA, F-OA, and TOA signals (Figure 4c), equal to 183 μm^2^/s for B-OA (broad signal at 5.55 ppm), 549 μm^2^/s for F-OA (multiplet at 5.51 ppm), and 855 μm^2^/s for TOA (triplet at 2.43 ppm; Figure 4c). The measured *D*_*t*_ value of pristine OA (1111 μm^2^/s, equal to the literature datum^48^) and the *D*_*t*_ value calculated for InAs tetrapods with a size of 10 nm (65.9 μm^2^/s) indicated that 89% of OA was bound to the NC. At the same time, the olefin signal of F-OA at 5.51 ppm had a *D*_*t*_ (549 μm^2^/s) which corresponded to the mean value between that of the pristine OA and the calculated value without any dynamic process at the surface (65.9 μm^2^/s), suggesting that the OA bound to InAs tetrapods underwent a slow exchange with the small amount of free OA present in solution. Conversely, the observed *D*_*t*_ value for TOA in tetrapods was slightly smaller than that of a pristine TOA (889 μm^2^/s, Figure S23), indicating that such a tertiary amine was only loosely bound to the NCs surface (the bound fraction of TOA was calculated to be only 3.5%) and underwent a fast exchange with the majority of free TOA.

**Figure 5:**
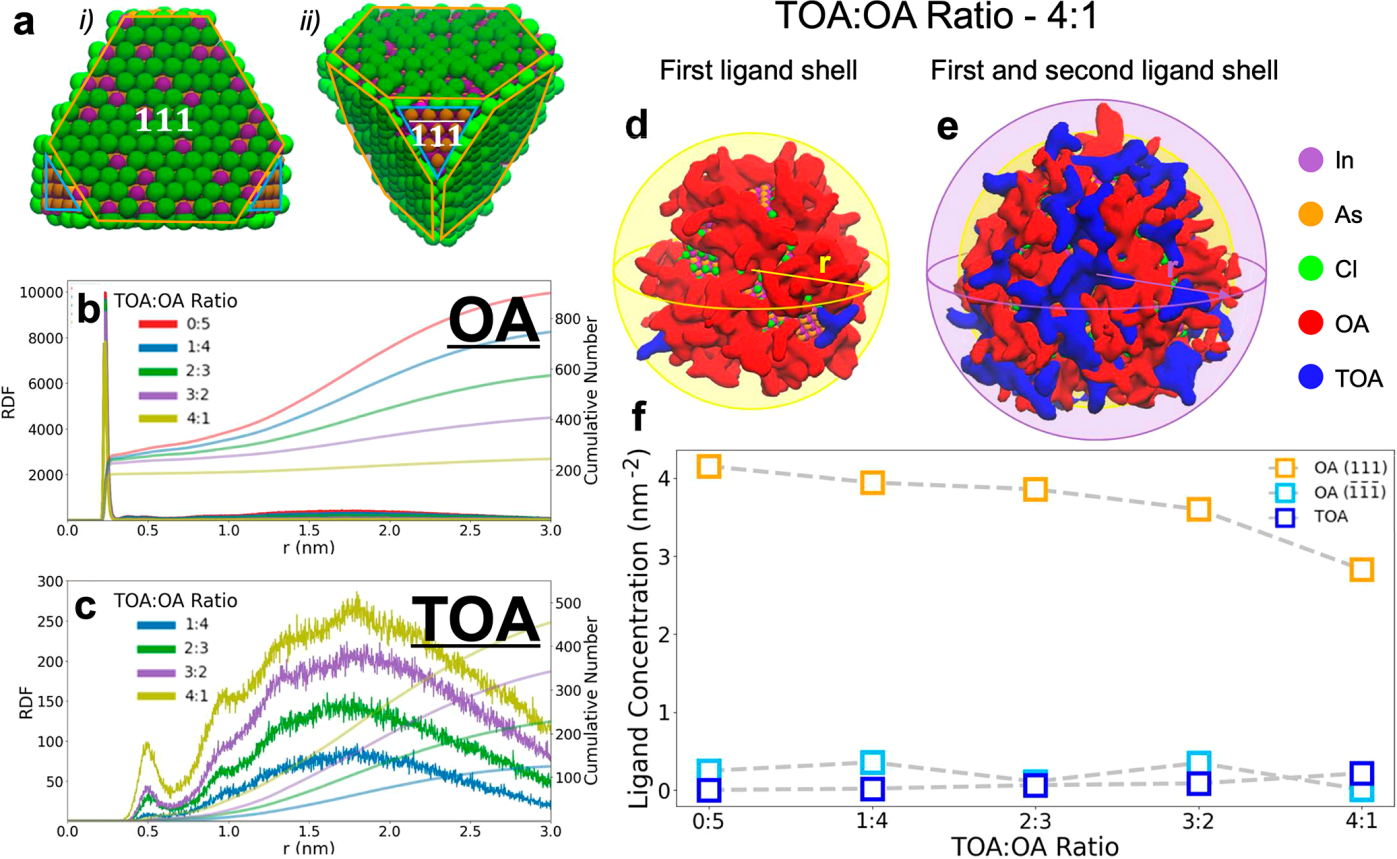
(a) Model systems of an InAs NC viewed from (111) facets (i, in orange) and (111) facets (ii, in light blue) as used in the MD simulations. (b, c) Radial distribution functions (RDFs) and cumulative number (CN) of ligands for OA (b) and TOA (c) molecules plotted at different TOA:OA volume ratios. (d, e) Representation of the first (d, at *r* = 3.0 Å) and second (e, at *r* = 6.8 Å) ligand shells in the model: OA molecules are depicted in red and TOA in blue. (f) Ligand concentrations of OA on (111) and (111) facets and TOA for all facets at different TOA:OA ratios.

To understand how the presence of TOA and OA affects InAs NCs growth and eventually leads to the formation of tetrapods, we performed classical MD simulations. We prepared a set of simulation boxes filled with one InAs NC, with stoichiometry In_889_As_776_ passivated by 339 Cl^–^ ions to maintain the system charge-balanced (Figure 5a, see the Experimental Section for details on the box preparation) and a number of OA and TOA molecules to satisfy the desired ratios (see Table S1). We performed MD simulations following the approach described in detail in the Experimental Section. The final MD trajectories were analyzed by computing the radial distribution functions (RDFs) between the ligands (OA and TOA) and the surface of the NC (Figure 5b,c), allowing also the determination of the number of bound ligands on each facet and therefore of the ligand densities (ligands per nm^2^). The OA and TOA plots display common features at different concentrations: a sharp and intense OA peak at a distance of ∼3.0 Å from the surface (Figure 5b) of the NC and a second, weaker one produced by TOA molecules at ∼5.0 Å from the surface (Figure 5c). The RDF peaks reflect the strengths of the binding interactions between the ligands on the NC surface, highlighting the presence of two “binding” spheres (Figure 5d,e): a first one, where OA molecules physically occupy the empty ligand sites, featuring a strong bond that persists even after the OA concentration is reduced; a second one, in which the TOA molecules are dominant, but the peak is very shallow, indicating a weak bond with the NC surface. The weak binding of TOA persists also at high TOA concentrations, suggesting that the steric hindrance provided by the octylic tails of TOA molecules results in an unfavorable access to the surface. The integration of the RDF provides an estimate for the number of ligands present at a given distance from the surface, which in the first peak turns out to be the actual coordination number to the NC surface. In Table S1, we noticed that in the presence of sole OA (ratio TOA:OA 0:5), about 28% of all OA molecules available in the simulation box were bound to the NC surface, the large majority on the (111) facets with a negligible contribution to the As-rich (111). This corresponds to a ligand coverage of 4.15 ligands/nm^2^ (Figure 5f) on the (111) facets. By reducing the OA concentration, the fraction of bound OA increased up to 77%; however, in absolute terms the number of OA ligands on the NC surface was reduced, with a ligand concentration that steadily dropped to 2.83 ligands/nm^2^ (Figure 5f). In contrast, the total number of TOA ligands bound to the NC surface was very small, ranging from 0.02 ligands/nm^2^ for TOA:OA 1:4 to 0.20 ligands/nm^2^ for the TOA:OA 4:1.

Overall, the MD simulations were in close agreement with our NMR analyses and allowed us to infer how classical tetrahedral- and tetrapod-shaped InAs form. A high OA ligand density (i.e., TOA:OA < 2:3) corresponds to a strong stabilization of the (111) facets, which leads to a final tetrahedral structure. On the other hand, at high TOA:OA ratios (i.e., 4:1) the OA ligand density on the (111) facets is halved, indicating a higher instability for these facets, which are susceptible to grow as arms and transform the NC into a tetrapod.

## Conclusion

In this work, we studied how surfactants different from the “classical” oleylamine (OA) influence the synthesis of InAs nanocrystals (NCs) via the amino-As route. Combinations of trioctylamine (TOA) and OA were found not only to enable a good control over the size and size distribution of InAs NCs but also to form tetrapods when employed in specific ratios (TOA:OA equal to 4:1). These tetrapods featured a cubic crystal structure, a core size of ∼2.5 nm, and arm lengths of 5–6 nm (with the arms growing along the ⟨111⟩ directions). Interestingly, our k·p calculations revealed that the bandgap of InAs tetrapods is not affected by the length of their arms when these are longer than ∼3 nm. This explains why, even if featuring a broad size distribution (in terms of arm lengths), the absorption peak of our InAs tetrapods was narrow (HWHM of 85 meV, which is among the smallest value reported for InAs colloidal nanostructures). Our MD simulations, in agreement with NMR analysis, revealed that while OA can strongly bind to (111) InAs NC facets, TOA can only weakly bind to the NCs surface (most likely on the edges and corners) as a consequence of its steric hindrance. Therefore, when employing high TOA:OA volume ratios InAs NCs can grow along the ⟨111⟩ directions eventually forming tetrapods. Our study provides new insights into the growth of InAs NCs via the amino-As route. Our results suggest that a pondered use of combinations of ligands could be another viable way to grow larger InAs NCs with good control over their size and size distribution.

## Supporting Information Available

Synthesis optimization with different ligand combinations, XRD patterns and TEM images of products made with sole TOP, DOA, TOA, oleic, and octadecylphosphonic acids, TEM pictures of InAs made with different TOA:OA ratios, PL decay curve and HAADF STEM of InAs tetrapods, MD parameters, k·p description, NMR analyses (PDF)

## References

[ref1] Wijaya H.; Darwan D.; Lim K. R. G.; Wang T.; Khoo K. H.; Tan Z.-K. Large-Stokes-Shifted Infrared-Emitting InAs-In(Zn)P-ZnSe-ZnS Giant-Shell Quantum Dots by One-Pot Continuous-Injection Synthesis. Chem. Mater. 2019, 31, 2019–2026. 10.1021/acs.chemmater.8b05023.

[ref2] Enright M. J.; Jasrasaria D.; Hanchard M. M.; Needell D. R.; Phelan M. E.; Weinberg D.; McDowell B. E.; Hsiao H.-W.; Akbari H.; Kottwitz M.; Potter M. M.; Wong J.; Zuo J.-M.; Atwater H. A.; Rabani E.; Nuzzo R. G. Role of Atomic Structure on Exciton Dynamics and Photoluminescence in NIR Emissive InAs/InP/ZnSe Quantum Dots. J. Phys. Chem. C 2022, 126, 7576–7587. 10.1021/acs.jpcc.2c01499.

[ref3] Goossens S.; Navickaite G.; Monasterio C.; Gupta S.; Piqueras J. J.; Pérez R.; Burwell G.; Nikitskiy I.; Lasanta T.; Galán T.; Puma E.; Centeno A.; Pesquera A.; Zurutuza A.; Konstantatos G.; Koppens F. Broadband Image Sensor Array Based on Graphene-Cmos Integration. Nat. Photonics 2017, 11, 366–371. 10.1038/nphoton.2017.75.

[ref4] Pradhan S.; Di Stasio F.; Bi Y.; Gupta S.; Christodoulou S.; Stavrinadis A.; Konstantatos G. High-Efficiency Colloidal Quantum Dot Infrared Light-Emitting Diodes Via Engineering at the Supra-Nanocrystalline Level. Nat. Nanotechnol. 2019, 14, 72–79. 10.1038/s41565-018-0312-y. 30510279

[ref5] Geiregat P.; Houtepen A. J.; Sagar L. K.; Infante I.; Zapata F.; Grigel V.; Allan G.; Delerue C.; Van Thourhout D.; Hens Z. Continuous-Wave Infrared Optical Gain and Amplified Spontaneous Emission at Ultralow Threshold by Colloidal HgTe Quantum Dots. Nat. Mater. 2018, 17, 35–42. 10.1038/nmat5000. 29035357

[ref6] Medintz I. L.; Uyeda H. T.; Goldman E. R.; Mattoussi H. Quantum Dot Bioconjugates for Imaging, Labelling and Sensing. Nat. Mater. 2005, 4, 435–446. 10.1038/nmat1390. 15928695

[ref7] Whitworth G. L.; Dalmases M.; Taghipour N.; Konstantatos G. Solution-Processed PbS Quantum Dot Infrared Laser with Room-Temperature *T*unable Emission in the Optical Telecommunications Window. Nat. Photonics 2021, 15, 738–742. 10.1038/s41566-021-00878-9. 34616485 PMC7611770

[ref8] Keuleyan S.; Lhuillier E.; Guyot-Sionnest P. Synthesis of Colloidal HgTe Quantum Dots for Narrow Mid-IR Emission and Detection. J. Am. Chem. Soc. 2011, 133, 16422–16424. 10.1021/ja2079509. 21942339

[ref9] Pietryga J. M.; Schaller R. D.; Werder D.; Stewart M. H.; Klimov V. I.; Hollingsworth J. A. Pushing the Band Gap Envelope: Mid-Infrared Emitting Colloidal PbSe Quantum Dots. J. Am. Chem. Soc. 2004, 126, 11752–11753. 10.1021/ja047659f. 15382884

[ref10] McDonald S. A.; Konstantatos G.; Zhang S.; Cyr P. W.; Klem E. J. D.; Levina L.; Sargent E. H. Solution-Processed PbS Quantum Dot Infrared Photodetectors and Photovoltaics. Nat. Mater. 2005, 4, 138–142. 10.1038/nmat1299. 15640806

[ref11] George E.; Pecht M. Rohs Compliance in Safety and Reliability Critical Electronics. Microelectron. Reliab. 2016, 65, 1–7. 10.1016/j.microrel.2016.07.150.

[ref12] Puttlitz K. J.; Galyon G. T. Impact of the RoHS Directive on High-Performance Electronic Systems. In Lead-Free Electronic Solders; Springer: 2006; pp 347–365.

[ref13] Gensch C.-O.; Baron Y.; Blepp M.; Deubzer O. Assistance to the Commission on Technological Socio-Economic and Cost-Benefit Assessment Related to Exemptions from the Substance Restrictions in Electrical and Electronic Equipment (Rohs Directive). Öko-Institut eV, Freiburg, Germany, 2016.

[ref14] Darwan D.; Lim L. J.; Wang T.; Wijaya H.; Tan Z.-K. Ultra-Confined Visible-Light-Emitting Colloidal Indium Arsenide Quantum Dots. Nano Lett. 2021, 21, 5167–5172. 10.1021/acs.nanolett.1c01223. 34096315

[ref15] Franke D.; Harris D. K.; Xie L.; Jensen K. F.; Bawendi M. G. The Unexpected Influence of Precursor Conversion Rate in the Synthesis of III-V Quantum Dots. Angew. Chem. 2015, 127, 14507–14511. 10.1002/ange.201505972. 26437711

[ref16] Schileo G.; Grancini G. Lead or No Lead? Availability, Toxicity, Sustainability and Environmental Impact of Lead-Free Perovskite Solar Cells. J. Mater. Chem. C 2021, 9, 67–76. 10.1039/D0TC04552G.

[ref17] Battaglia D.; Peng X. Formation of High Quality InP and InAs Nanocrystals in a Noncoordinating Solvent. Nano Lett. 2002, 2, 1027–1030. 10.1021/nl025687v.

[ref18] Franke D.; Harris D. K.; Chen O.; Bruns O. T.; Carr J. A.; Wilson M. W. B.; Bawendi M. G. Continuous Injection Synthesis of Indium Arsenide Quantum Dots Emissive in the Short-Wavelength Infrared. Nat. Commun. 2016, 7, 12749 10.1038/ncomms12749. 27834371 PMC5114595

[ref19] Bahmani Jalali H.; De Trizio L.; Manna L.; Di Stasio F. Indium Arsenide Quantum Dots: An Alternative to Lead-Based Infrared Emitting Nanomaterials. Chem. Soc. Rev. 2022, 51, 9861–9881. 10.1039/D2CS00490A. 36408788 PMC9743785

[ref20] De Franco M.; Zhu D.; Asaithambi A.; Prato M.; Charalampous E.; Christodoulou S.; Kriegel I.; De Trizio L.; Manna L.; Bahmani Jalali H.; Di Stasio F. Near-Infrared Light-Emitting Diodes Based on Rohs-Compliant InAs/ZnSe Colloidal Quantum Dots. ACS Energy Lett. 2022, 7, 3788–3790. 10.1021/acsenergylett.2c02070. 36398094 PMC9664446

[ref21] Peng X.; Wickham J.; Alivisatos A. P. Kinetics of II-VI and III-V Colloidal Semiconductor Nanocrystal Growth: “Focusing” of Size Distributions. J. Am. Chem. Soc. 1998, 120, 5343–5344. 10.1021/ja9805425.

[ref22] Xie R.; Chen K.; Chen X.; Peng X. InAs/InP/ZnSe Core/Shell/Shell Quantum Dots as Near-Infrared Emitters: Bright, Narrow-Band, Non-Cadmium Containing, and Biocompatible. Nano Res. 2008, 1, 457–464. 10.1007/s12274-008-8048-x. 20631914 PMC2902876

[ref23] Das A.; Shamirian A.; Snee P. T. Arsenic Silylamide: An Effective Precursor for Arsenide Semiconductor Nanocrystal Synthesis. Chem. Mater. 2016, 28, 4058–4064. 10.1021/acs.chemmater.6b01577.

[ref24] Guzelian A. A.; Banin U.; Kadavanich A. V.; Peng X.; Alivisatos A. P. Colloidal Chemical Synthesis and Characterization of InAs Nanocrystal Quantum Dots. Appl. Phys. Lett. 1996, 69, 1432–1434. 10.1063/1.117605.

[ref25] Kim S.-W.; Zimmer J. P.; Ohnishi S.; Tracy J. B.; Frangioni J. V.; Bawendi M. G. Engineering InAs_x_P_1-x_/InP/ZnSe III-V Alloyed Core/Shell Quantum Dots for the Near-Infrared. J. Am. Chem. Soc. 2005, 127, 10526–10532. 10.1021/ja0434331. 16045339

[ref26] Harris D. K.; Bawendi M. G. Improved Precursor Chemistry for the Synthesis of III-V Quantum Dots. J. Am. Chem. Soc. 2012, 134, 20211–20213. 10.1021/ja309863n. 23228014 PMC3535303

[ref27] Zimmer J. P.; Kim S.-W.; Ohnishi S.; Tanaka E.; Frangioni J. V.; Bawendi M. G. Size Series of Small Indium Arsenide-Zinc Selenide Core-Shell Nanocrystals and Their Application to in Vivo Imaging. J. Am. Chem. Soc. 2006, 128, 2526–2527. 10.1021/ja0579816. 16492023 PMC2753875

[ref28] Sagar L. K.; Bappi G.; Johnston A.; Chen B.; Todorović P.; Levina L.; Saidaminov M. I.; García de Arquer F. P.; Hoogland S.; Sargent E. H. Single-Precursor Intermediate Shelling Enables Bright, Narrow Line Width InAs/InZnP-Based QD Emitters. Chem. Mater. 2020, 32, 2919–2925. 10.1021/acs.chemmater.9b05110.

[ref29] Wells R. L.; Aubuchon S. R.; Kher S. S.; Lube M. S.; White P. S. Synthesis of Nanocrystalline Indium Arsenide and Indium Phosphide from Indium (III) Halides and Tris (Trimethylsilyl) Pnicogens. Synthesis, Characterization, and Decomposition Behavior of I_3_In·P(SiMe_3_)_3_. Chem. Mater. 1995, 7, 793–800. 10.1021/cm00052a027.

[ref30] Tamang S.; Lee S.; Choi H.; Jeong S. Tuning Size and Size Distribution of Colloidal InAs Nanocrystals Via Continuous Supply of Prenucleation Clusters on Nanocrystal Seeds. Chem. Mater. 2016, 28, 8119–8122. 10.1021/acs.chemmater.6b03585.

[ref31] Asor L.; Liu J.; Ossia Y.; Tripathi D. C.; Tessler N.; Frenkel A. I.; Banin U. InAs Nanocrystals with Robust p-Type Doping. Adv. Funct. Mater. 2021, 31, 2007456 10.1002/adfm.202007456.

[ref32] Grigel V.; Dupont D.; De Nolf K.; Hens Z.; Tessier M. D. InAs Colloidal Quantum Dots Synthesis Via Aminopnictogen Precursor Chemistry. J. Am. Chem. Soc. 2016, 138, 13485–13488. 10.1021/jacs.6b07533. 27701864

[ref33] Zhao T.; Oh N.; Jishkariani D.; Zhang M.; Wang H.; Li N.; Lee J. D.; Zeng C.; Muduli M.; Choi H.-J.; et al. General Synthetic Route to High-Quality Colloidal III-V Semiconductor Quantum Dots Based on Pnictogen Chlorides. J. Am. Chem. Soc. 2019, 141, 15145–15152. 10.1021/jacs.9b06652. 31496238

[ref34] Ginterseder M.; Franke D.; Perkinson C. F.; Wang L.; Hansen E. C.; Bawendi M. G. Scalable Synthesis of InAs Quantum Dots Mediated through Indium Redox Chemistry. J. Am. Chem. Soc. 2020, 142, 4088–4092. 10.1021/jacs.9b12350. 32073841

[ref35] Tietze R.; Panzer R.; Starzynski T.; Guhrenz C.; Frenzel F.; Würth C.; Resch-Genger U.; Weigand J. J.; Eychmüller A. Synthesis of NIR-emitting InAs-based Core/Shell Quantum Dots with the Use of Tripyrazolylarsane as Arsenic Precursor. Part. Part. Syst. Charact. 2018, 35, 1800175 10.1002/ppsc.201800175.

[ref36] Srivastava V.; Dunietz E.; Kamysbayev V.; Anderson J. S.; Talapin D. V. Monodisperse InAs Quantum Dots from Aminoarsine Precursors: Understanding the Role of Reducing Agent. Chem. Mater. 2018, 30, 3623–3627. 10.1021/acs.chemmater.8b01137.

[ref37] Srivastava V.; Janke E. M.; Diroll B. T.; Schaller R. D.; Talapin D. V. Facile, Economic and Size-Tunable Synthesis of Metal Arsenide Nanocrystals. Chem. Mater. 2016, 28, 6797–6802. 10.1021/acs.chemmater.6b03501.

[ref38] Zhu D.; Bellato F.; Bahmani Jalali H.; Di Stasio F.; Prato M.; Ivanov Y. P.; Divitini G.; Infante I.; De Trizio L.; Manna L. ZnCl_2_ Mediated Synthesis of InAs Nanocrystals with Aminoarsine. J. Am. Chem. Soc. 2022, 144, 10515–10523. 10.1021/jacs.2c02994. 35648676 PMC9204758

[ref39] Kim T.; Park S.; Jeong S. Diffusion Dynamics Controlled Colloidal Synthesis of Highly Monodisperse InAs Nanocrystals. Nat. Commun. 2021, 12, 3013 10.1038/s41467-021-23259-w. 34021149 PMC8140152

[ref40] Rosen E. L.; Buonsanti R.; Llordes A.; Sawvel A. M.; Milliron D. J.; Helms B. A. Exceptionally Mild Reactive Stripping of Native Ligands from Nanocrystal Surfaces by Using Meerwein’s Salt. Angew. Chem., Int. Ed. 2012, 51, 684–689. 10.1002/anie.201105996. 22147424

[ref41] Wu P. S. C.; Otting G. Rapid Pulse Length Determination in High-Resolution NMR. J. Magn. Reson. 2005, 176, 115–119. 10.1016/j.jmr.2005.05.018. 15972263

[ref42] Jeener J.; Meier B. H.; Bachmann P.; Ernst R. R. Investigation of Exchange Processes by Two-Dimensional NMR Spectroscopy. J. Chem. Phys. 1979, 71, 4546–4553. 10.1063/1.438208.

[ref43] Wagner R.; Berger S. Gradient-Selected NOESY—a Fourfold Reduction of the Measurement Time for the NOESY Experiment. J. Magn. Reson., Ser. A 1996, 123, 119–121. 10.1006/jmra.1996.0222. 8980072

[ref44] Wu D. H.; Chen A. D.; Johnson C. S. An Improved Diffusion-Ordered Spectroscopy Experiment Incorporating Bipolar-Gradient Pulses. J. Magn. Reson., Ser. A 1995, 115, 260–264. 10.1006/jmra.1995.1176.

[ref45] Abraham M. J.; Murtola T.; Schulz R.; Páll S.; Smith J. C.; Hess B.; Lindahl E. Gromacs: High Performance Molecular Simulations through Multi-Level Parallelism from Laptops to Supercomputers. SoftwareX 2015, 1–2, 19–25. 10.1016/j.softx.2015.06.001.

[ref46] Essmann U.; Perera L.; Berkowitz M. L.; Darden T.; Lee H.; Pedersen L. G. A Smooth Particle Mesh Ewald Method. J. Chem. Phys. 1995, 103, 8577–8593. 10.1063/1.470117.

[ref47] Yesselman J. D.; Price D. J.; Knight J. L.; Brooks III C. L. Match: An Atom-Typing Toolset for Molecular Mechanics Force Fields. J. Comput. Chem. 2012, 33, 189–202. 10.1002/jcc.21963. 22042689 PMC3228871

[ref48] Leemans J.; Dümbgen K. C.; Minjauw M. M.; Zhao Q.; Vantomme A.; Infante I.; Detavernier C.; Hens Z. Acid-Base Mediated Ligand Exchange on Near-Infrared Absorbing, Indium-Based III-V Colloidal Quantum Dots. J. Am. Chem. Soc. 2021, 143, 4290–4301. 10.1021/jacs.0c12871. 33710882

[ref49] Kim Y.; Choi H.; Lee Y.; Koh W.-k.; Cho E.; Kim T.; Kim H.; Kim Y.-H.; Jeong H. Y.; Jeong S. Tailored Growth of Single-Crystalline InP Tetrapods. Nat. Commun. 2021, 12, 4454 10.1038/s41467-021-24765-7. 34294721 PMC8298524

[ref50] Kim S.; Park S.; Kim M.; Jeong S. Synthesis of Single-Crystalline InP Tetrapod Nanocrystals Via Addition of ZnCl_2_. Bull. Korean Chem. Soc. 2023, 44, 483 10.1002/bkcs.12684.

[ref51] Hassinen A.; Moreels I.; de Mello Donegá C.; Martins J. C.; Hens Z. Nuclear Magnetic Resonance Spectroscopy Demonstrating Dynamic Stabilization of CdSe Quantum Dots by Alkylamines. J. Phys. Chem. Lett. 2010, 1, 2577–2581. 10.1021/jz100781h.

[ref52] Mourdikoudis S.; Liz-Marzán L. M. Oleylamine in Nanoparticle Synthesis. Chem. Mater. 2013, 25, 1465–1476. 10.1021/cm4000476.

[ref53] De Roo J.; Ibáñez M.; Geiregat P.; Nedelcu G.; Walravens W.; Maes J.; Martins J. C.; Van Driessche I.; Kovalenko M. V.; Hens Z. Highly Dynamic Ligand Binding and Light Absorption Coefficient of Cesium Lead Bromide Perovskite Nanocrystals. ACS Nano 2016, 10, 2071–2081. 10.1021/acsnano.5b06295. 26786064

[ref54] Almeida G.; Ashton O. J.; Goldoni L.; Maggioni D.; Petralanda U.; Mishra N.; Akkerman Q. A.; Infante I.; Snaith H. J.; Manna L. The Phosphine Oxide Route toward Lead Halide Perovskite Nanocrystals. J. Am. Chem. Soc. 2018, 140, 14878–14886. 10.1021/jacs.8b08978. 30358392 PMC6438589

[ref55] Hens Z.; Martins J. C. A Solution NMR Toolbox for Characterizing the Surface Chemistry of Colloidal Nanocrystals. Chem. Mater. 2013, 25, 1211–1221. 10.1021/cm303361s.

[ref56] Iannone F.; Ambrosino F.; Bracco G.; De Rosa M.; Funel A.; Guarnieri G.; Migliori S.; Palombi F.; Ponti G.; Santomauro G.; Procacci P. Cresco Enea Hpc Clusters: A Working Example of a Multifabric GPFS Spectrum Scale Layout. In 2019 International Conference on High Performance Computing Simulation (HPCS), 2019; pp 1051–1052.

