# Supplementary material for: Colloidal InAs Tetrapods: Impact of Surfactants on the Shape Control
Source: J Am Chem Soc. 2023 Aug 8;145(33):18329–39. doi: 10.1021/jacs.3c03906 (PMC10450814; doi:10.1021/jacs.3c03906)
Supplement: Supplementary file 1 — ja3c03906_si_001.pdf [file ja3c03906_si_001.pdf]

Supporting Information for:

Colloidal InAs Tetrapods: Impact of Surfactants on the Shape Control

Zheming Liu[a,b], Roberta Pascazio[a,b], Luca Goldoni[a,c,d], Daniela Maggioni[e], Dongxu Zhu[a], Yurii P. Ivanov[f], Giorgio Divitini[f], Jordi Llusar Camarelles[g], Houman Bahmani Jalali[a,h], Ivan Infante*[g,i], Luca De Trizio*[j] and Liberato Manna*[a]

[a] Nanochemistry, [c] Analytical Chemistry, [d] Materials Characterization, [f] Electron Spectroscopy and Nanoscopy, [h] Photonic Nanomaterials and [j] Chemistry Facility, Istituto Italiano di Tecnologia, Via Morego 30, 16163 Genova, Italy

[b] Dipartimento di Chimica e Chimica Industriale, Università di Genova, 16146 Genova, Italy

[e] Dipartimento di Chimica, Università degli Studi di Milano, Via Golgi 19, 20133 Milano, Italy

[g] BCMaterials, Basque Center for Materials, Applications, and Nanostructures, UPV/EHU Science Park, Leioa 48940, Spain

[i] Ikerbasque Basque Foundation for Science Bilbao 48009, Spain

Synthesis of "standard" InAs NCs with sole OA

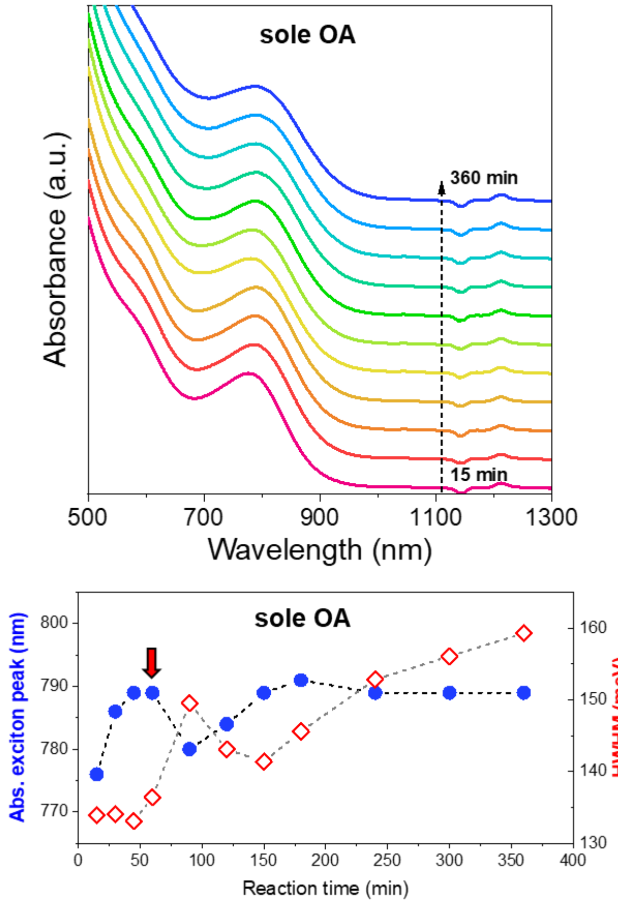

Figure S1. The synthesis of InAs NCs with sole OA was allowed to proceed for 360 min. Aliquots were taken at different reaction times. The absorption curves and the corresponding position of the first exciton peak and HWHM are reported in the top and bottom panels, respectively. The red arrow indicates the time up to which the NCs grew in the size focusing regime. Such reaction time (60 min) was employed to produce the "standard" NC sample reported in the main text.

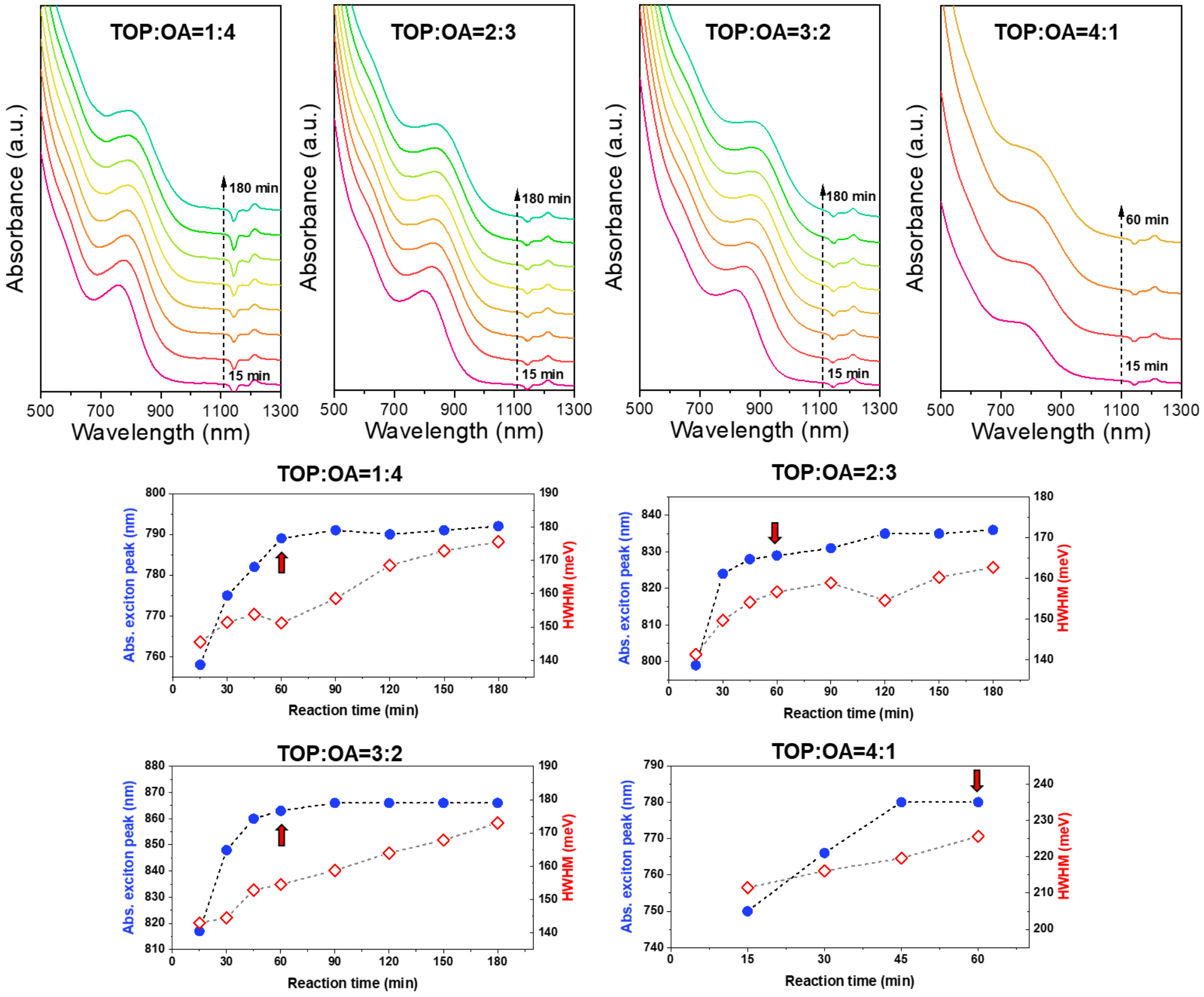

Figure S2. The syntheses of InAs NCs with 1:4, 2:3 and 3:2 TOP:OA volume ratios were allowed to proceed for 180 min while the synthesis based on the TOP:OA ratio of 4:1 was stopped after 60 min. Aliquots were taken at different reaction times. The absorption curves and the corresponding position of the first exciton peak and HWHM are reported in the top and bottom panels, respectively. The red arrows indicate the time up to which the NCs grew in the size focusing regime. Such reaction time (60 min) was employed to synthesize the "TOP:OA" NC samples reported in the main text.

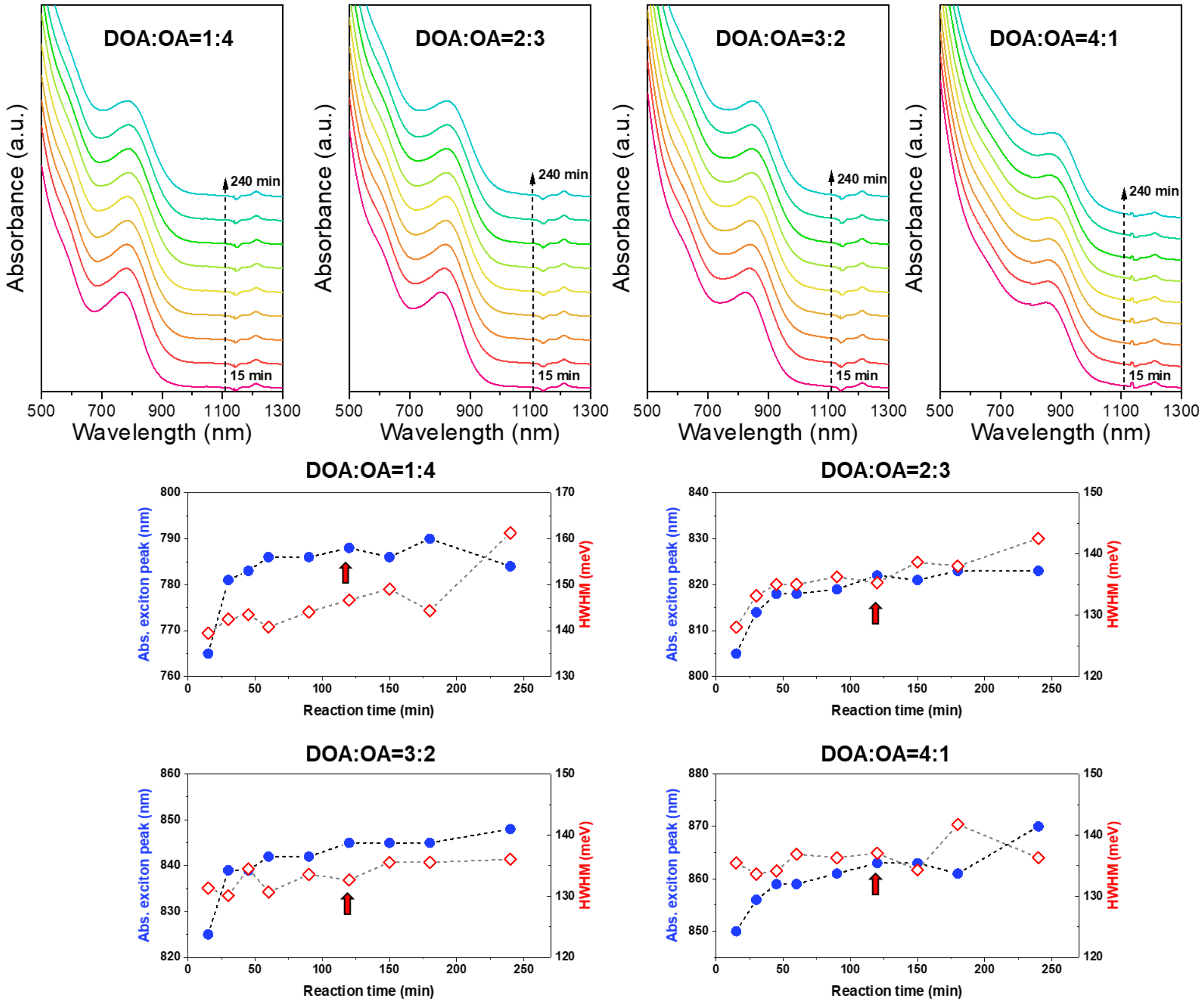

Figure S3. The syntheses of InAs NCs with different DOA:OA volume ratios were allowed to proceed for 240 min. Aliquots were taken at different reaction times. The absorption curves and the corresponding position of the first exciton peak and HWHM are reported in the top and bottom panels, respectively. The red arrows indicate the time up to which the NCs grew in the size focusing regime. Such reaction time (120 min) was employed to synthesize the "DOA:OA" NC samples reported in the main text.

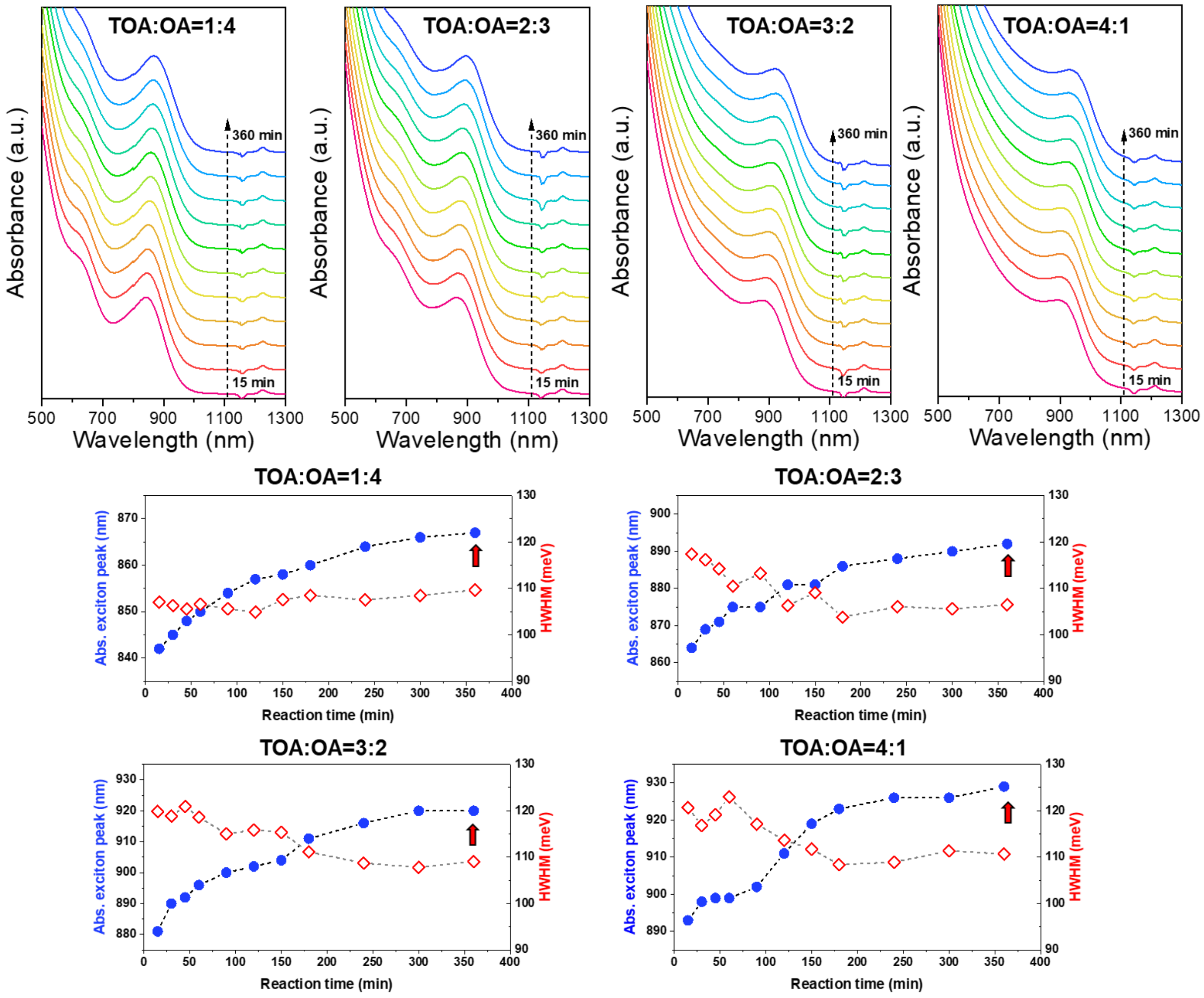

Figure S4. The syntheses of InAs NCs with different TOA:OA ratios were allowed to proceed for 360 min. Aliquots were taken at different reaction times. The absorption curves and the corresponding position of the first exciton peak and HWHM are reported in the top and bottom panels, respectively. The red arrows indicate the time up to which the NCs grew in the size focusing regime. Such reaction time (360 min) was employed to synthesize the "TOA:OA" NC samples reported in the main text.

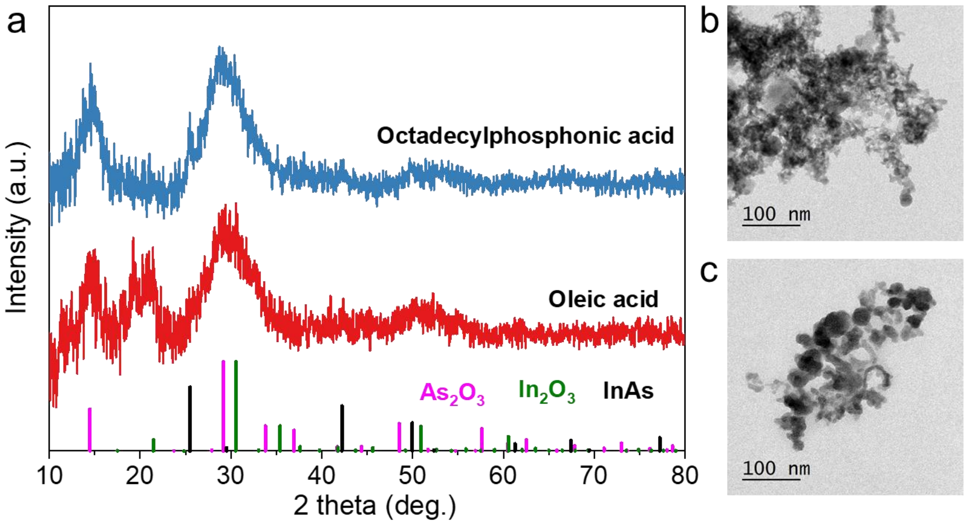

Figure S5. a) XRD patterns of products obtained when employing oleic or octadecylphosphonic acids together with the bulk reflections of As_2O_3 (COD number 96-41-3571), In_2O_3 (ICSD number 169432) and cubic InAs (ICSD number 24518) (reaction time 1h). TEM images of products obtained by using (b) octadecylphosphonic acid or (c) oleic acid.

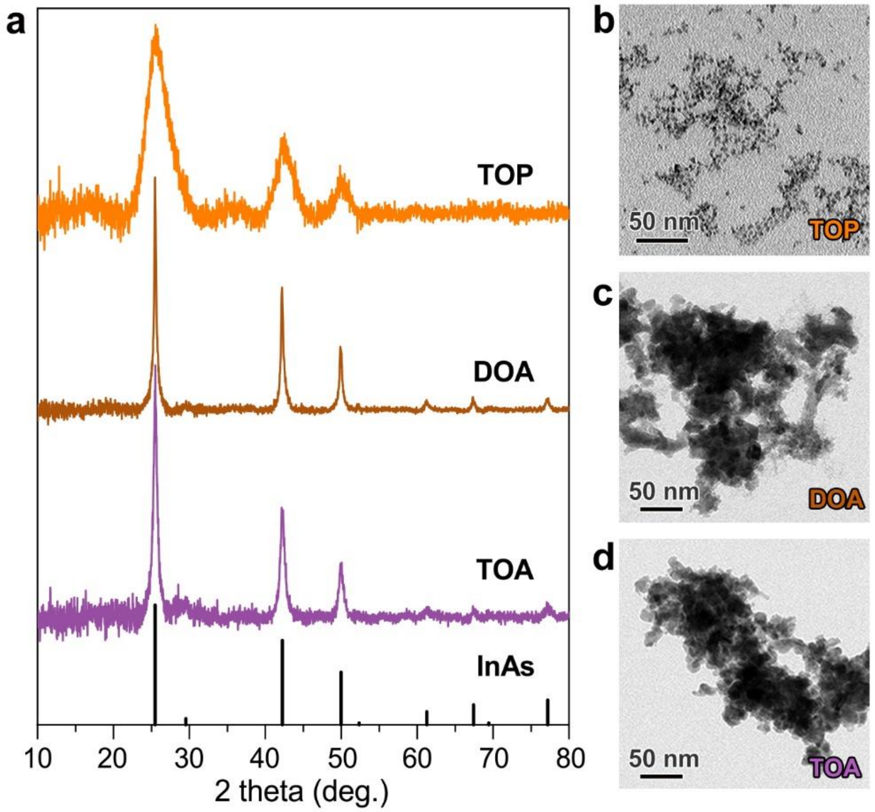

Figure S6. InAs NC samples made by employing only TOP, DOA or TOA (reaction time 1h): a) XRD patterns togheter with the bulk reflections of cubic InAs (ICSD number 24518), (b-d) TEM images.

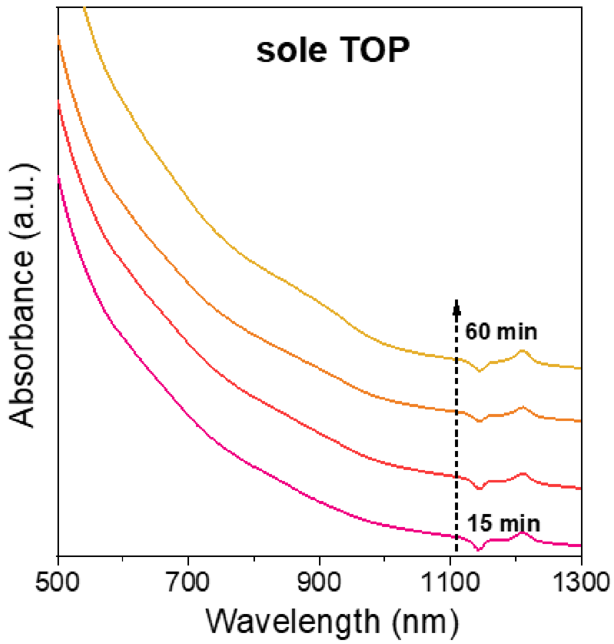

Figure S7. Optical absorption spectra of InAs NCs made with only TOP ligands.

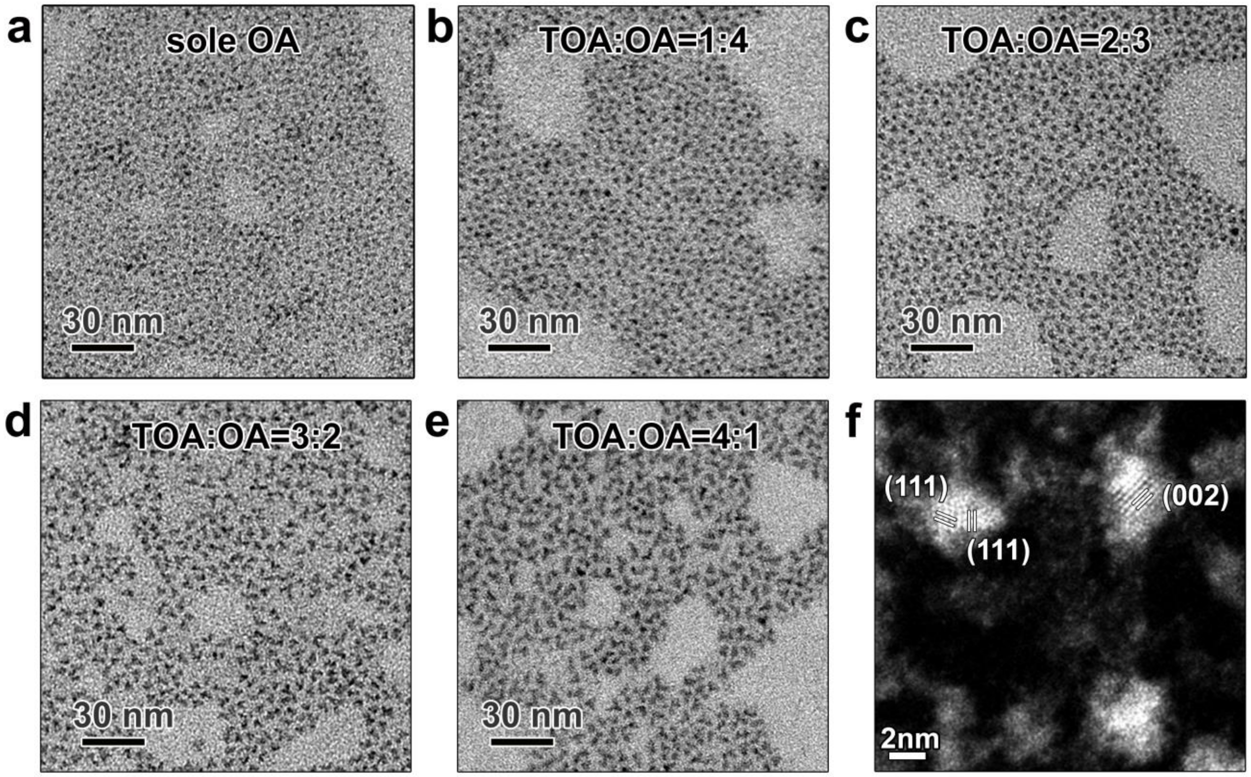

Figure S8. TEM images of InAs NCs synthesized by using (a) only OA or combinations of TOA and OA at TOA:OA volume ratios of (b) 1:4, (c) 2:3, (d) 3:2 and (e) 4:1. (f) HAADF HRSTEM image of InAs NCs synthesized with sole OA. The two NCs at the top of the figure are both aligned along the [110] zone axis.

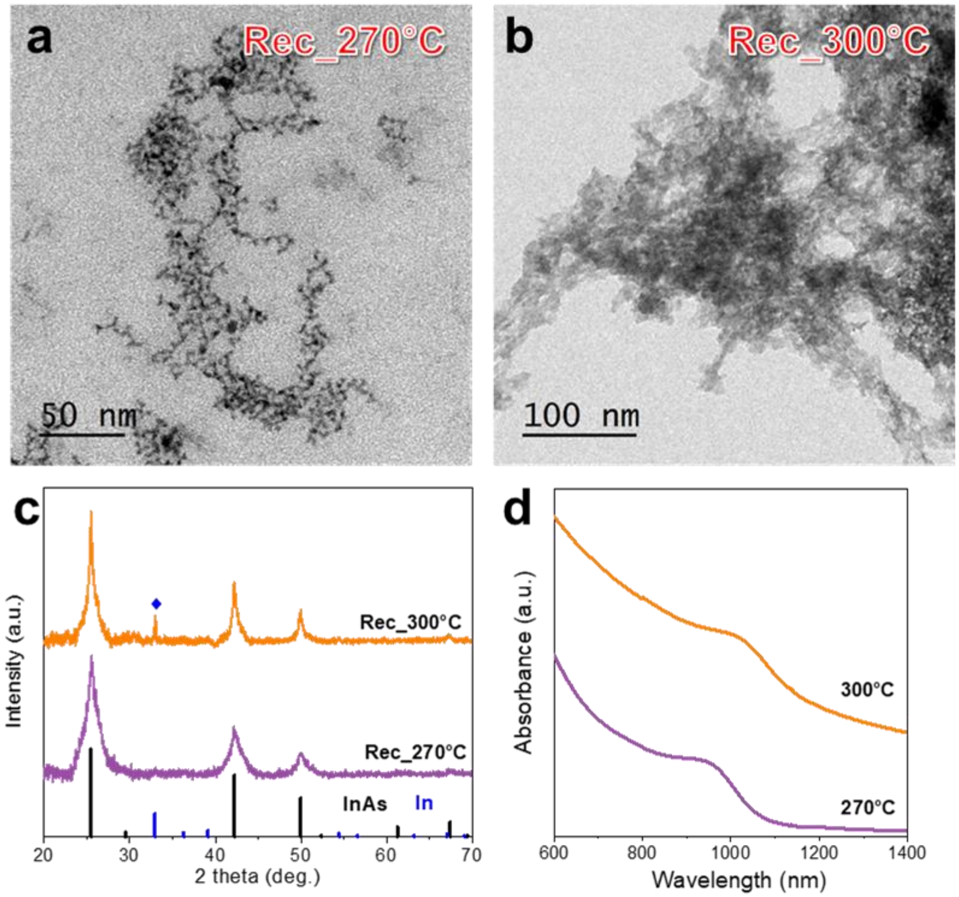

Figure S9. Synthesis of InAs NCs performed at either 270 °C or 300 °C with reaction time fixed at 15 min (severe aggregation at 30 min). In both syntheses the injection temperature of amino-As and DMEA-AlH_3 was 240 °C. (a, b) TEM images, (c) XRD patterns with the bulk reflections of InAs (ICSD number 24518) and In (ICSD number 171679) and (d) absorption spectra.

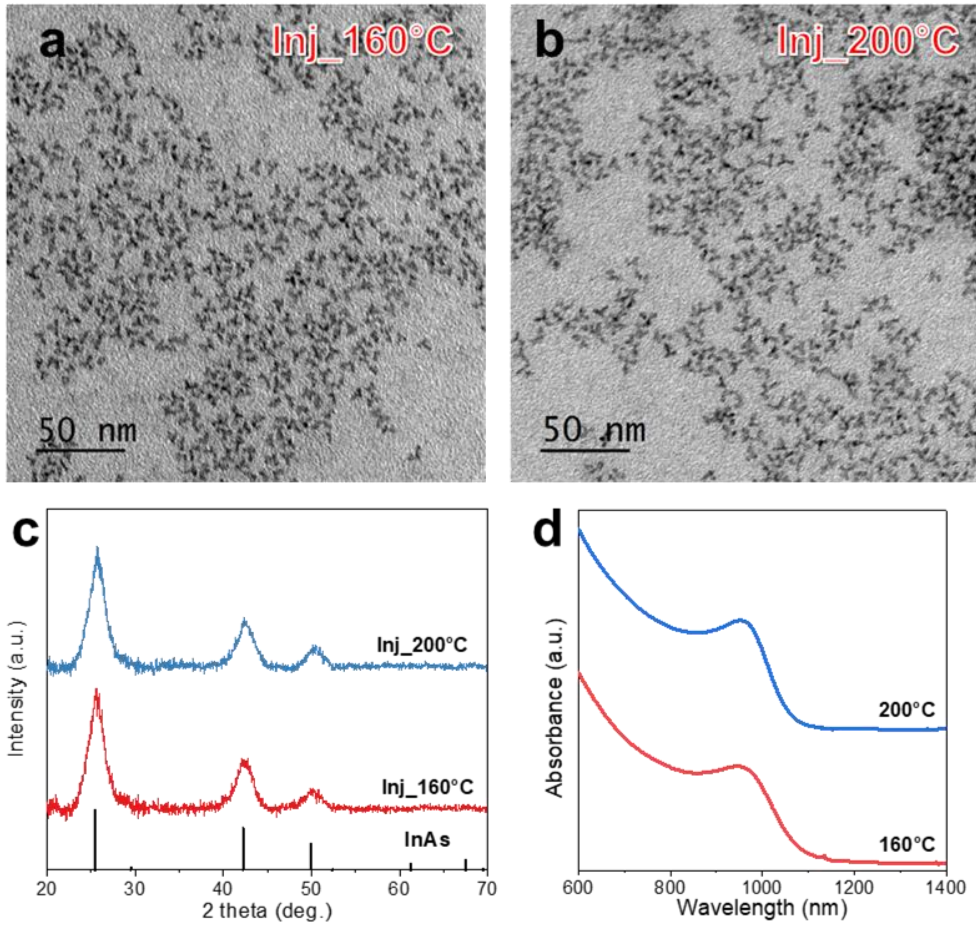

Figure S10. Synthesis of InAs NCs in which the injection temperature of amino-As and DMEA-AlH_3 was set to 160 °C or 200 °C. The reaction temperature was 240 °C and the reaction was allowed to run for 360 min in both cases. (a, b) TEM images, (c) XRD patterns with the bulk reflections of InAs (ICSD number 24518) and (d) absorption spectra.

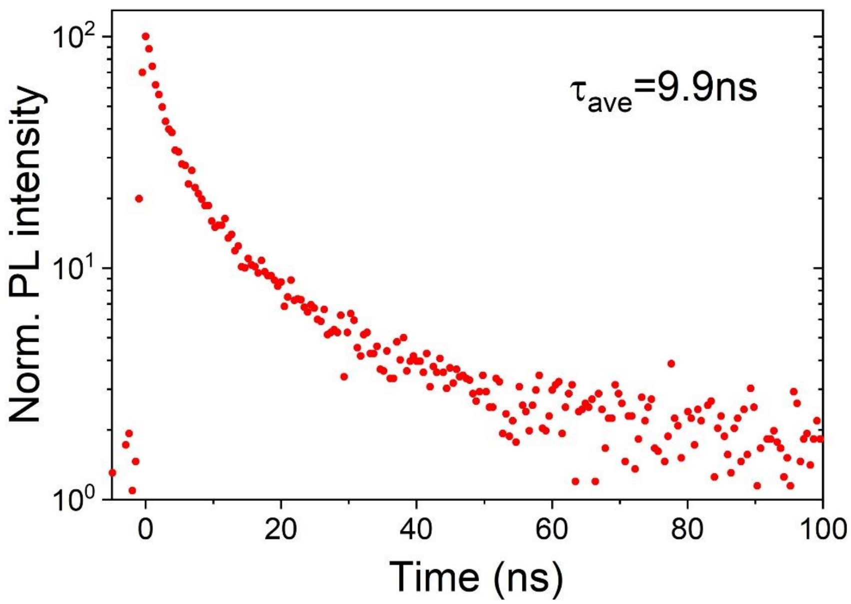

Figure S11. PL decay curves of tetrapod shaped InAs NCs.

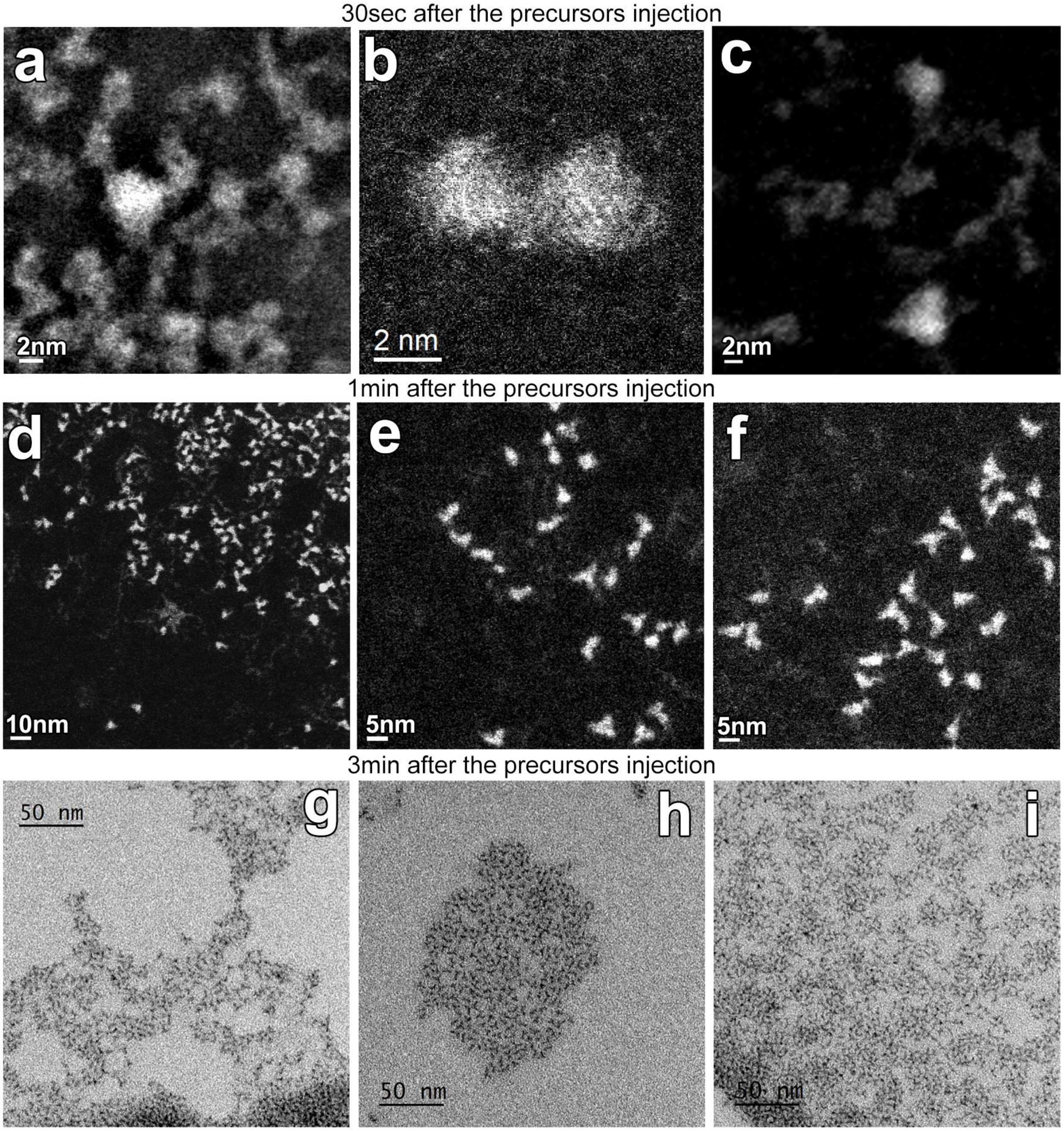

Figure S12. HAADF-STEM images of the NCs synthesized 30 sec (a-c) and 1 min (d-f) after the injection of the As precursor. g-i) TEM images of the product after 3min from the injection of the As precursor.

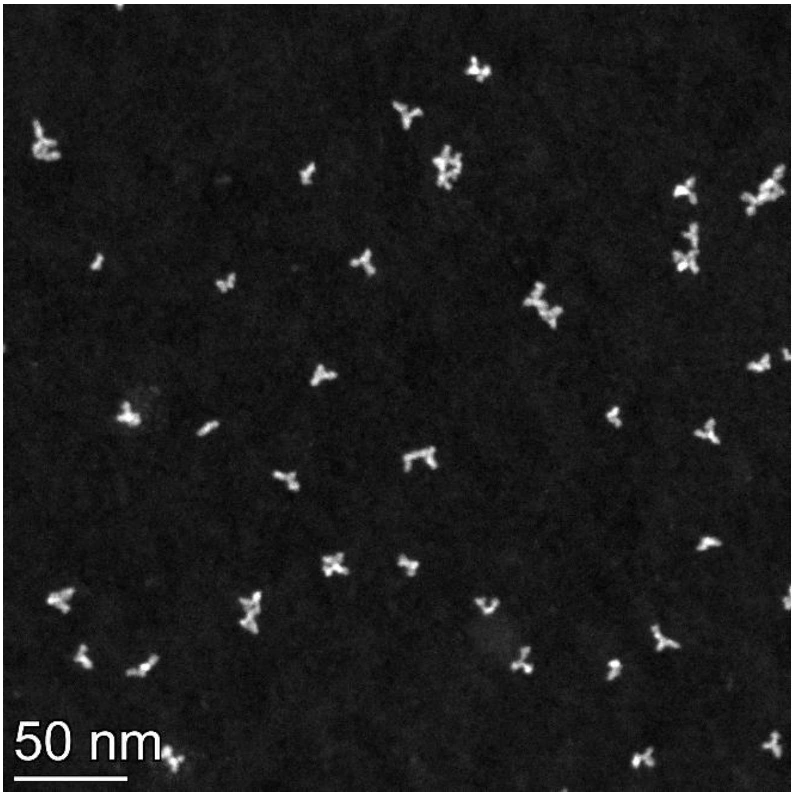

Figure S13. HAADF-STEM image of tetrapod InAs NCs.

Classical Molecular Dynamics (MD).

Table S1. Ligand Quantities and Concentrations in Simulation Boxes.

**TOA:OA Volume Ratio**	Ligands in the Simulation Box		Ligands on NC Surface				Ligand Concentration (nm^{-2})			
	TOA	OA	TOA	OA			TOA	OA		
			Total	Total	(111)	$(\overline{111})$	Total	Total	(111)	$(\overline{111})$
0:5	-	924	-	258	257	1	-	3.87	4.15	0.25
1:4	131	750	1	246	244	2	0.02	3.69	3.95	0.35
2:3	263	575	4	239	238	1	0.06	3.59	3.86	0.11
3:2	394	401	6	224	222	2	0.08	3.37	3.60	0.34
4:1	525	226	14	175	175	0	0.21	2.63	2.83	0.01

Table S2. Force Field Parameters.

<table>
<tr><th colspan="8">Non-Bonded Parameters</th></tr>
<tr><th colspan="8">Charge (e)</th></tr>
<tr><th colspan="2" rowspan="2">Core</th><th colspan="2" rowspan="2">Solvent</th><th colspan="4">Ligands</th></tr>
<tr><th colspan="2">TOA</th><th colspan="2">OA</th></tr>
<tr><td></td><td></td><td></td><td></td><td>N301</td><td>-0.63</td><td>N321</td><td>-0.86</td></tr>
<tr><td>In</td><td>1.11</td><td></td><td></td><td>C321</td><td>-0.18</td><td>C321</td><td>-0.18</td></tr>
<tr><td>As</td><td>-1.11</td><td>C262</td><td>0.0</td><td>C323</td><td>-0.03</td><td>C322</td><td>0.10</td></tr>
<tr><td>Cl</td><td>-0.37</td><td>C261</td><td>-0.115</td><td></td><td></td><td>C2D1</td><td>-0.15</td></tr>
<tr><td></td><td></td><td>C331</td><td>-0.27</td><td>C331</td><td>-0.27</td><td>C331</td><td>-0.27</td></tr>
<tr><td></td><td></td><td></td><td></td><td></td><td></td><td>HGA4</td><td>0.15</td></tr>
<tr><td></td><td></td><td>HGA3</td><td>0.09</td><td>HGA3</td><td>0.09</td><td>HGA3</td><td>0.09</td></tr>
<tr><td></td><td></td><td></td><td></td><td>HGA2</td><td>0.09</td><td>HGA2</td><td>0.09</td></tr>
<tr><td></td><td></td><td>HG61</td><td>0.12</td><td></td><td></td><td>HPA2</td><td>0.29</td></tr>
<tr><th colspan="4">Sigma (nm)</th><th colspan="4">Epsilon (kJ mol^{-1})</th></tr>
<tr><td colspan="2">In In</td><td colspan="2">0.410</td><td colspan="2">In In</td><td colspan="2">2.506</td></tr>
<tr><td colspan="2">As In</td><td colspan="2">0.297</td><td colspan="2">As In</td><td colspan="2">1.800</td></tr>
<tr><td colspan="2">Cl In</td><td colspan="2">0.257</td><td colspan="2">Cl In</td><td colspan="2">1.543</td></tr>
<tr><td colspan="2">As As</td><td colspan="2">0.424</td><td colspan="2">As As</td><td colspan="2">1.293</td></tr>
<tr><td colspan="2">As Cl</td><td colspan="2">0.398</td><td colspan="2">As Cl</td><td colspan="2">1.108</td></tr>
<tr><td colspan="2">Cl Cl</td><td colspan="2">0.391</td><td colspan="2">Cl Cl</td><td colspan="2">0.950</td></tr>
<tr><td colspan="2">HPA2 In</td><td colspan="2">0.234</td><td colspan="2">HPA2 In</td><td colspan="2">0.324</td></tr>
<tr><td colspan="2">HPA2 As</td><td colspan="2">0.327</td><td colspan="2">HPA2 As</td><td colspan="2">0.233</td></tr>
<tr><td colspan="2">HPA2 Cl</td><td colspan="2">0.189</td><td colspan="2">HPA2 Cl</td><td colspan="2">0.199</td></tr>
<tr><td colspan="2">N321 In</td><td colspan="2">0.266</td><td colspan="2">N321 In</td><td colspan="2">0.793</td></tr>
<tr><td colspan="2">N321 As</td><td colspan="2">0.376</td><td colspan="2">N321 As</td><td colspan="2">0.570</td></tr>
<tr><td colspan="2">N321 Cl</td><td colspan="2">0.359</td><td colspan="2">N321 Cl</td><td colspan="2">0.488</td></tr>
<tr><th colspan="8">Bonded Parameters</th></tr>
<tr><th colspan="4">Bonds</th><th colspan="4">Angles</th></tr>
<tr><th colspan="2"></th><th>b_0 (nm)</th><th>k_b (kJ nm^{-2})</th><th colspan="2"></th><th>θ_0 (deg)</th><th>k_θ (kJ deg^{-2})</th></tr>
</table>

C321 C321	0.153	1.86E+05	C321 C321 C321	113.6	4.88E+02
C321 HGA2	0.111	2.59E+05	HGA2 C321 C321	110.1	2.22E+02
C321 C331	0.153	1.86E+05	HGA2 C321 HGA2	109	2.97E+02
C321 N321	0.147	2.20E+05	C321 C321 C331	115	4.85E+02
C2D1 C321	0.150	3.05E+05	C321 C321 N321	112.2	2.68E+02
C331 HGA3	0.111	2.69E+05	C321 C321 C2D1	112.2	2.68E+02
HPA2 N321	0.101	3.79E+05	HGA2 C321 C331	110.1	2.90E+02
C2D1 C2D1	0.134	3.68E+05	C321 C331 HGA3	110.1	2.90E+02
C2D1 HGA4	0.110	3.02E+05	HGA2 C321 N321	109.5	2.71E+02
C321 C322	0.153	1.86E+05	C321 N321 HPA2	112.1	3.43E+02
C322 HGA2	0.111	2.59E+05	C2D1 C2D1 C321	123.5	4.02E+02
C322 N321	0.147	2.20E+05	HGA4 C2D1 C321	116	3.35E+02
C323 N301	0.147	2.20E+05	C2D1 C321 HGA2	111.5	3.77E+02
C321 C323	0.153	1.86E+05	HGA3 C331 HGA3	108.4	2.97E+02
C323 HGA2	0.111	2.59E+05	HPA2 N321 HPA2	105.85	2.47E+02
C261 C261	0.138	2.55E+05	HGA4 C2D1 C2D1	119.5	4.35E+02
C261 HG61	0.108	2.85E+05	C322 C321 C321	113.6	4.88E+02
C261 C262	0.138	2.55E+05	C322 C321 C322	113.6	4.88E+02
C262 C331	0.149	1.92E+05	HGA2 C322 C321	110.1	2.22E+02
			HGA2 C321 C322	110.1	2.22E+02
			HGA2 C322 HGA2	109	2.97E+02
			C321 C322 N321	112.2	2.68E+02
			HGA2 C322 N321	109.5	2.71E+02
			C322 N321 HPA2	112.1	3.43E+02
			C321 C323 N301	113.5	5.86E+02
			HGA2 C323 N301	109.5	2.71E+02
			C323 N301 C323	115.6	2.93E+02
			HGA2 C321 C323	110.1	2.22E+02
			HGA2 C323 C321	110.1	2.22E+02

			C323 C321 C321	113.6	4.88E+02
			C321 C321 C323	113.6	4.88E+02
			HGA2 C323 HGA2	109	2.97E+02
			C261 C261 C261	120	3.35E+02
			C261 C261 C262	120	3.35E+02
			C331 C262 C261	120	3.83E+02
			C261 C261 HG61	120	2.51E+02
			C262 C261 HG61	120	2.51E+02
			C262 C331 HGA3	107.5	4.13E+02
**Dihedral Angles**					
	**φ_0 (deg)**	**k_φ (kJ deg^{-2})**		**φ_0 (deg)**	**k_φ (kJ deg^{-2})**
C321 C321 C321 C321	180	6.27E-01	HGA2 C322 C321 C321	0	8.16E-01
C321 C321 C321 C321	0	3.96E-01	HGA2 C321 C322 C321	0	8.16E-01
C321 C321 C321 C321	0	4.71E-01	HGA2 C321 C321 C322	0	8.16E-01
C321 C321 C321 C321	0	2.70E-01	HGA2 C322 C321 HGA2	0	9.20E-01
HGA2 C321 C321 C321	0	8.16E-01	HGA2 C321 C322 HGA2	0	9.20E-01
HGA2 C321 C321 HGA2	0	9.20E-01	C321 C321 C322 N321	0	7.11E-01
C321 C321 C321 C331	180	3.40E-01	C321 C321 C322 N321	180	2.09E-01
C321 C321 C321 C331	0	4.53E-01	C321 C321 C322 N321	180	5.86E-01
C321 C321 C321 C331	0	8.53E-01	HGA2 C321 C322 N321	0	8.16E-01
C321 C321 C321 C331	0	6.30E-01	C321 C322 N321 HPA2	0	6.69E-01

C321 C321 C321 N321	0	7.11E-01	HGA2 C322 N321 HPA2	0	4.18E-02
C321 C321 C321 N321	180	2.09E-01	C321 C321 C323 N301	0	8.37E-01
C321 C321 C321 N321	180	5.86E-01	HGA2 C321 C323 N301	0	8.16E-01
C321 C321 C321 C2D1	0	7.11E-01	C321 C323 N301 C323	180	7.53E-01
C321 C321 C321 C2D1	180	2.09E-01	C321 C323 N301 C323	0	2.72E-01
C321 C321 C321 C2D1	180	5.86E-01	C321 C323 N301 C323	0	1.26E-01
HGA2 C321 C321 C331	0	7.53E-01	C321 C323 N301 C323	0	3.14E+00
C321 C321 C331 HGA3	0	6.69E-01	HGA2 C323 N301 C323	0	4.18E-01
HGA2 C321 C321 N321	0	8.16E-01	HGA2 C321 C321 C323	0	8.16E-01
C321 C321 N321 HPA2	0	6.69E-01	HGA2 C323 C321 C321	0	8.16E-01
HGA2 C321 C321 C2D1	0	8.16E-01	HGA2 C323 C321 HGA2	0	9.20E-01
C321 C321 C2D1 C2D1	180	2.51E+00	HGA2 C321 C323 HGA2	0	9.20E-01
C321 C321 C2D1 HGA4	0	5.02E-01	C323 C321 C321 C321	180	6.27E-01
HGA2 C321 C331 HGA3	0	6.69E-01	C321 C321 C321 C323	180	6.27E-01
HGA2 C321 N321 HPA2	0	4.18E-02	C323 C321 C321 C321	0	3.96E-01
C321 C2D1 C2D1 C321	180	3.56E+01	C321 C321 C321 C323	0	3.96E-01
C321 C2D1 C2D1 C321	180	1.88E+00	C323 C321 C321 C321	0	4.71E-01
HGA4 C2D1 C2D1 C321	180	4.18E+00	C321 C321 C321 C323	0	4.71E-01

C2D1 C2D1 C321 HGA2	180	1.26E+00	C323 C321 C321 C321	0	2.70E-01
HGA4 C2D1 C321 HGA2	0	0.00E+00	C321 C321 C321 C323	0	2.70E-01
HGA4 C2D1 C2D1 HGA4	180	4.18E+00	C261 C261 C261 C261	180	1.30E+01
C322 C321 C321 C321	180	6.27E-01	C262 C261 C261 C261	180	1.30E+01
C321 C321 C321 C322	180	6.27E-01	HG61 C261 C261 C261	180	1.76E+01
C322 C321 C321 C321	0	3.96E-01	HG61 C261 C261 C262	180	1.76E+01
C321 C321 C321 C322	0	3.96E-01	HG61 C261 C262 C261	180	1.76E+01
C322 C321 C321 C321	0	4.71E-01	HGA3 C331 C262 C261	0	8.37E-03
C321 C321 C321 C322	0	4.71E-01	C331 C262 C261 C261	180	1.30E+01
C322 C321 C321 C321	0	2.70E-01	C331 C262 C261 HG61	180	1.00E+01
C321 C321 C321 C322	0	2.70E-01	HG61 C261 C261 HG61	180	1.00E+01

K·P model description

The choice of the 1-(heavy-hole) band Hamiltonian for holes was based on the following criteria: i) the heavy-hole band is clearly split away from the light-hole band. In InAs NCs, the optical absorption value of the heavy-hole band is ~200 nm (~ 500 meV) away from the light-hole band (see Figure 1a of the main text).[1] InAs tetrapods reported by Ginterseder et al. (see the supporting information of the reference paper)[2], similar to those synthesised in the present work, show comparable energy shifts. This energy difference between light-hole and heavy-hole optical absorption has also been observed experimentally in this work. ii) The system is strain-free. This means that the nature of the valence band maximum (VBM), which is usually the heavy hole band, should be preserved.[3] Moreover, the absence of strain yields negligible effects of the split-off valence band on the band-edge and their effective masses.[4] iii) Additionally, the spin-orbit splitting energy is relatively large, 0.38 eV (**Table S3**). This shifts downward the split-off valence band and, in turn, reduces the interaction with both the light-hole and the heavy-hole bands. Therefore, (i) and (ii) allow one to rule out the light-hole band and (iii) the split-off band from the analysis, consequently keeping only the heavy-hole band.

Hence, we could use, without loss of generality, one-band Hamiltonians for both electrons and holes. However, for the electrons, it must be considered that the conduction band minimum (CBM) is not only isotropic, but also non-parabolic.[5] To account for this non-parabolicity in the 1-band Hamiltonian for electrons, the electron effective mass m_e^* should turn into:

$$\frac{1}{m_e^*} \longrightarrow \gamma_c = \frac{1}{m_e^*} - \frac{E_p}{E_g}\left(\frac{E_g + {}^{2\Delta_{so}}/_{3}}{E_g + \Delta_{so}}\right) . \tag{1}$$

Here, m_e^* is the parabolic effective mass; E_p is the Kane parameter, which considers the conduction and valence bands interaction; E_g is the bulk bandgap energy; and Δ_{so} is the spin-orbit splitting energy. Therefore, the 1-band single-electron Hamiltonian is given by:

$$\hat{\mathcal{H}}_e = -\frac{\hbar^2}{2m_0}\gamma_c k^2 + E_g + V_{ext} , \tag{2}$$

where $\hbar$ is the Planck constant; m_0 is the free electron mass; k is the three-dimensional wavevector; and V_{ext} is a piecewise external potential that in our case will stand for an infinite potential barrier outside the system of study as a boundary condition and will be null within it.

For the hole case, since here we have used anisotropic effective masses, the 1-band single-hole Hamiltonian reads:

$$\hat{\mathcal{H}}_h = -\frac{\hbar^2}{2m_0}[k_z^2(\gamma_1 - 2\gamma_3) - k_\perp^2(\gamma_1 + \gamma_3)] + V_{ext} . \tag{3}$$

Here, γ_i are the modified Luttinger parameters, which can be directly derived from the usual Luttinger parameters γ_i^L.[6] In our particular case, the use of the modified Luttinger parameters is convenient so that the effects of the spin-orbit coupling, the interaction between the CBM and VBM, and the bandgap energy are considered. Therefore, the full Hamiltonian is as follows:

$$\hat{\mathcal{H}} = \hat{\mathcal{H}}_e + \hat{\mathcal{H}}_h \tag{4}$$

Role of the Coulomb interaction

Notice that here we did not implement the Coulomb interaction between electron and hole in (4), however we have performed direct Coulomb energies, just to know what is the order of magnitude of this interaction, taking the following integral, which is the direct Coulomb interaction integral J_{eh}:

$$J_{eh} = q_e \langle \psi_h | V | \psi_h \rangle \quad (5)$$

where q_e is the electron charge; ψ_h is the normalized ground state hole wavefunction; and V is the Coulomb potential. The latter has been calculated through the Poisson equation as follows:

$$-q_e |\psi_e|^2 = \varepsilon_0 \nabla \cdot (\varepsilon_r \nabla V) \quad (6)$$

Here, ψ_e is the normalized ground state electron wavefunction ε_0 and ε_r are the vacuum and relative permittivity, respectively. Note that in equation (6) the electron acts as a source of the interaction.

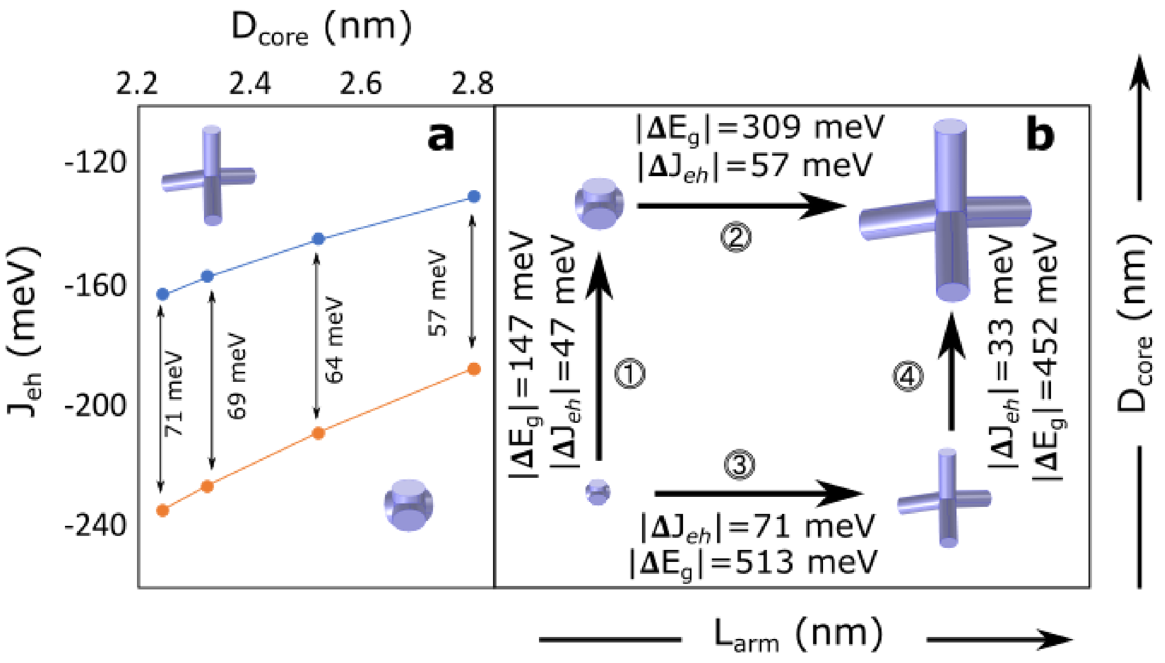

Figure S14. (a) Evolution of the direct Coulomb interaction J_{eh}, for a pseudo-truncated tetrahedral NC and a tetrapod, as the core size changes. (b) Energy shift comparison between quantum confinement, **|ΔE_g|** and direct Coulomb energies, **|ΔJ_{eh}|**; ① represents the system transition from the minimum to maximum core diameter pseudo-truncated tetrahedral NCs, ② from the maximum core diameter of a pseudo-truncated tetrahedral NC to a maximum size tetrapod (max. core diameter and arm lengths), ③ from the minimum size pseudo-truncated tetrahedral NC to minimum size tetrapod (min. core diameter and max. arm lengths) and ④ from the minimum to the maximum size tetrapods.

We have obtained lower (pseudo-truncated tetrahedral NC) and upper bounds (tetrapod of 5 nm arm length) for the direct Coulomb interaction energy. From Figure S14a, one can observe how the energetic shift that arises when moving from a pseudo-truncated tetrahedral NC to a tetrapod is relatively small (~71-57 meV when increasing the core diameter, Figure S14a). However, this shift, in the end, will be practically

dominated by quantum confinement effects by approximately a factor of 3, 5, 7 and 14, for system transitions ①, ②, ③ and ④, respectively (Figure S14b).

Table S3. Parameters

**Parabolic electron effective mass**	m_e^*	$0.023m_0$	Ref. [7]
Bulk band gap energy at Γ	E_g	0.42 eV	Ref. [8]
Kane parameter	E_p	22.2 eV	
Spin-orbit splitting energy	Δ_{so}	0.38 eV	
Luttinger parameters	γ_1^L	20	
	γ_3^L	9	
Modified Luttinger parameters	γ_1	6.3887	$\gamma_1 = \gamma_1^L - \frac{E_p}{3E_g+\Delta_{so}}$ Ref.[6]
	γ_3	2.1944	$\gamma_3 = \gamma_3^L - \frac{E_p}{6E_g+2\Delta_{so}}$ Ref.[6]

Nuclear Magnetic Resonance analysis

Preparation of standard InAs NCs. InAs NCs synthesized by sole OA were washed through the following steps: (1) the crude reaction solution (2 mL) was mixed with 6 mL ethanol and then was centrifuged at 4500 rpm for 5 min. (2) The precipitate was dispersed in 2 mL of anhydrous toluene. (3) 6 mL of ethanol was added to solution and then the mixture was centrifuged at 4500 rpm for 5 min; (4) the precipitate was re-dispersed in 1 mL of anhydrous toluene-d_8; (5) the solution was centrifuged at 5500 rpm for 5 min and the supernatant was collected for NMR measurements.

Preparation of tetrapod InAs NCs. InAs NCs synthesized by TOA and OA ratio of 4:1 were washed through the follow step: (1) the crude reaction solution (2 mL) was mixed with 1 mL anhydrous toluene and 30 mL ethyl acetate and then was centrifuged at 11000 rpm for 20 min. (2) The precipitate was dispersed in 1 mL of anhydrous toluene-d_8. (3) the solution was centrifuged at 5500 rpm for 5 min and the supernatant was collected for NMR measurement.

Both OA and TOA ligands were analysed via NMR spectroscopy. In this case the degassed ligands were directly dispersed in anhydrous toluene-d_8.

^{1}H-^{13}C HSQC (multiplicity edited Heteronuclear Single Quantum Coherence, "*hsqcedetgpsp.3*" pulse sequence of Bruker library) were acquired at 298 K by using the Bruker AvanceIII 600 MHz spectrometer (^{1}H at 600.13 MHz and ^{13}C at 150.92 MHz), equipped with 5 mm QCI cryoprobe, for pristine ligand structure elucidation, with the following parameters: 1 FID, 2048 digit points, 521 increments and $^{1}J_{CH}$ = 145 Hz, a spectral width of 13.02 ppm for ^{1}H and 165.00 ppm for ^{13}C, with a transmitter frequency offset of 6.40 and 75.0 ppm respectively.

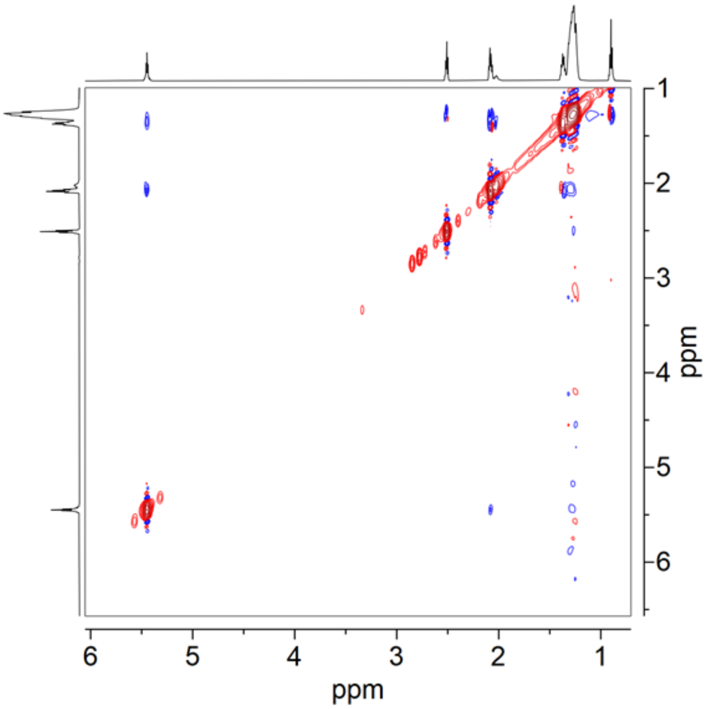

Figure S15. ^{1}H-^{1}H 2D NOESY spectrum of pristine OA in toluene-d_8 (298 K, 14.4 T, mixing time =300 ms), returns positive (blue) cross peaks that are typical of species with short correlation times (τ_c).

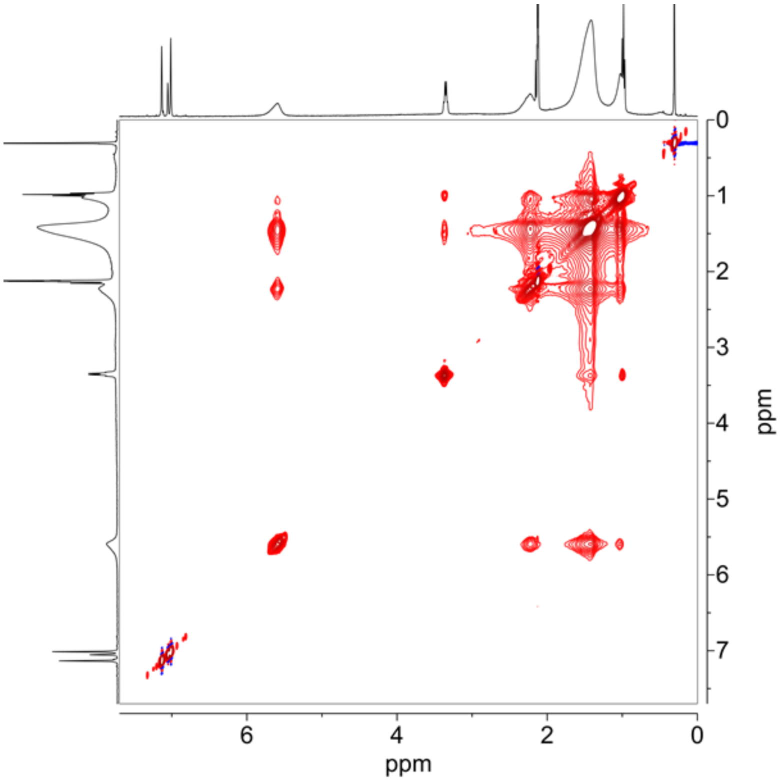

Figure S16. ^1H-^1H NOESY spectrum of "standard" InAs NCs solution in toluene-d_8 (298 K, 9.4 T, mixing time = 100 ms, RD = 2 s) returns negative (red) cross peaks that are typical of species with long correlation times (τ_c).

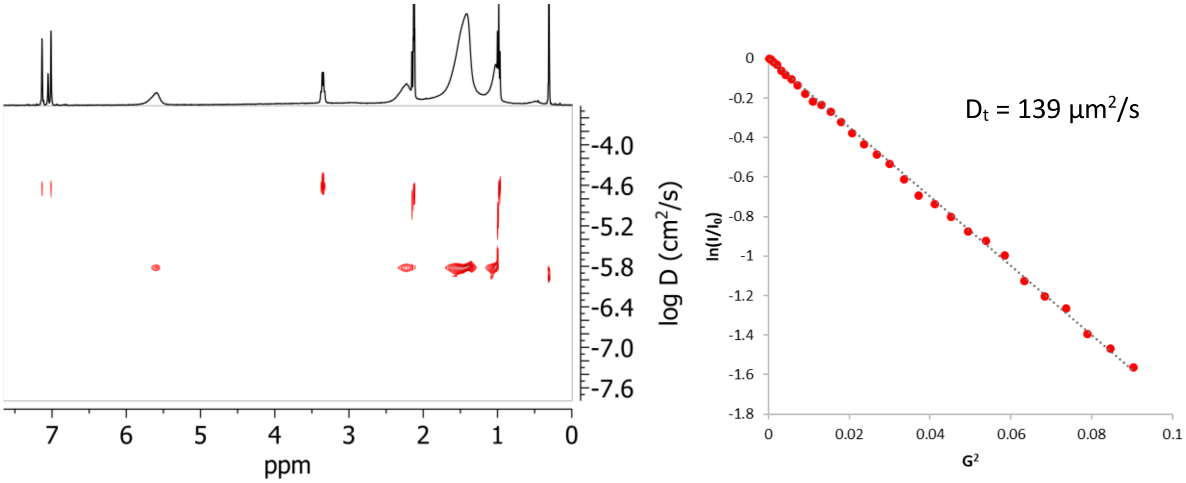

Figure S17. Left: ^1H DOSY spectrum of "standard" InAs NCs (298 K, 9.4 T, toluene-d_8, RD = 6 s); Right: Decay of a resonance of OA as a function of the squared gradient strength, whose slope affords the diffusion coefficient D_t.

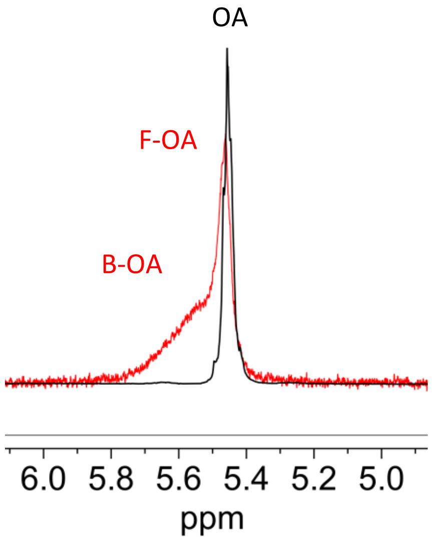

Figure S18. Comparison of the olefinic region of ^{1}H NMR spectra of pristine OA (black trace) and tetrapod InAs NCs (red trace) in toluene-d_8 at 300 K, 9.4 T.

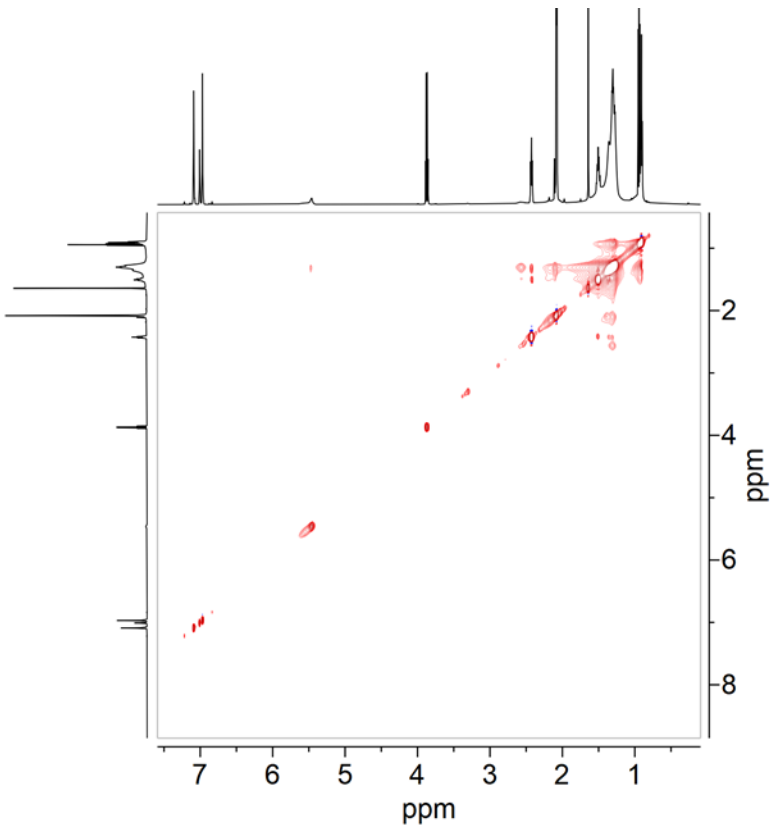

Figure S19. ^{1}H-NOESY 2D NMR experiment of tetrapod InAs NCs made by employing TOA and OA ligands (τ_{mix} = 100 ms, 14.1 T, 298K, toluene-d_8).

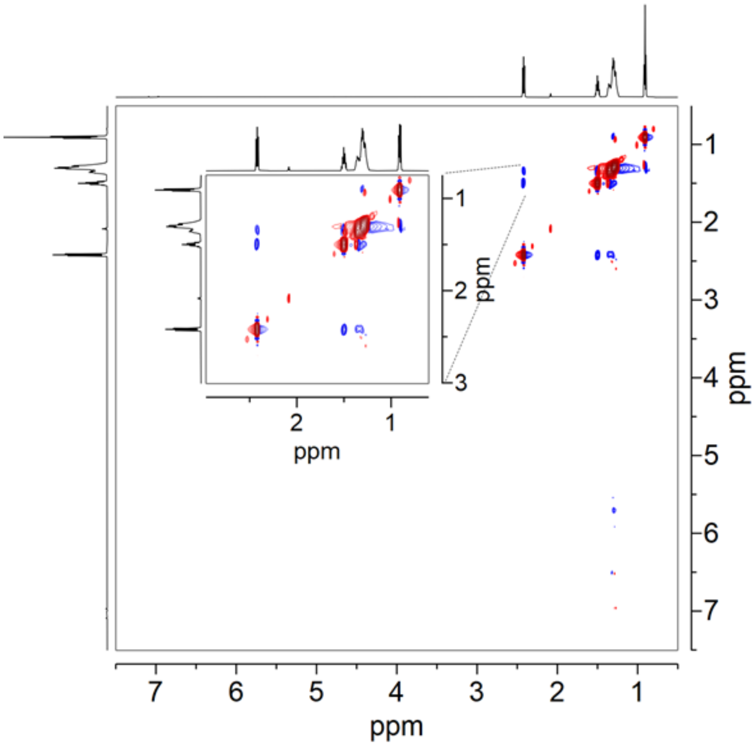

Figure S20. ^{1}H-^{1}H 2D NOESY spectrum of pristine TOA in toluene-d_8 (298 K, mixing time =300 ms, 14.1 T) returns positive (blue) cross peaks that are typical of species with short correlation times (τ_c).

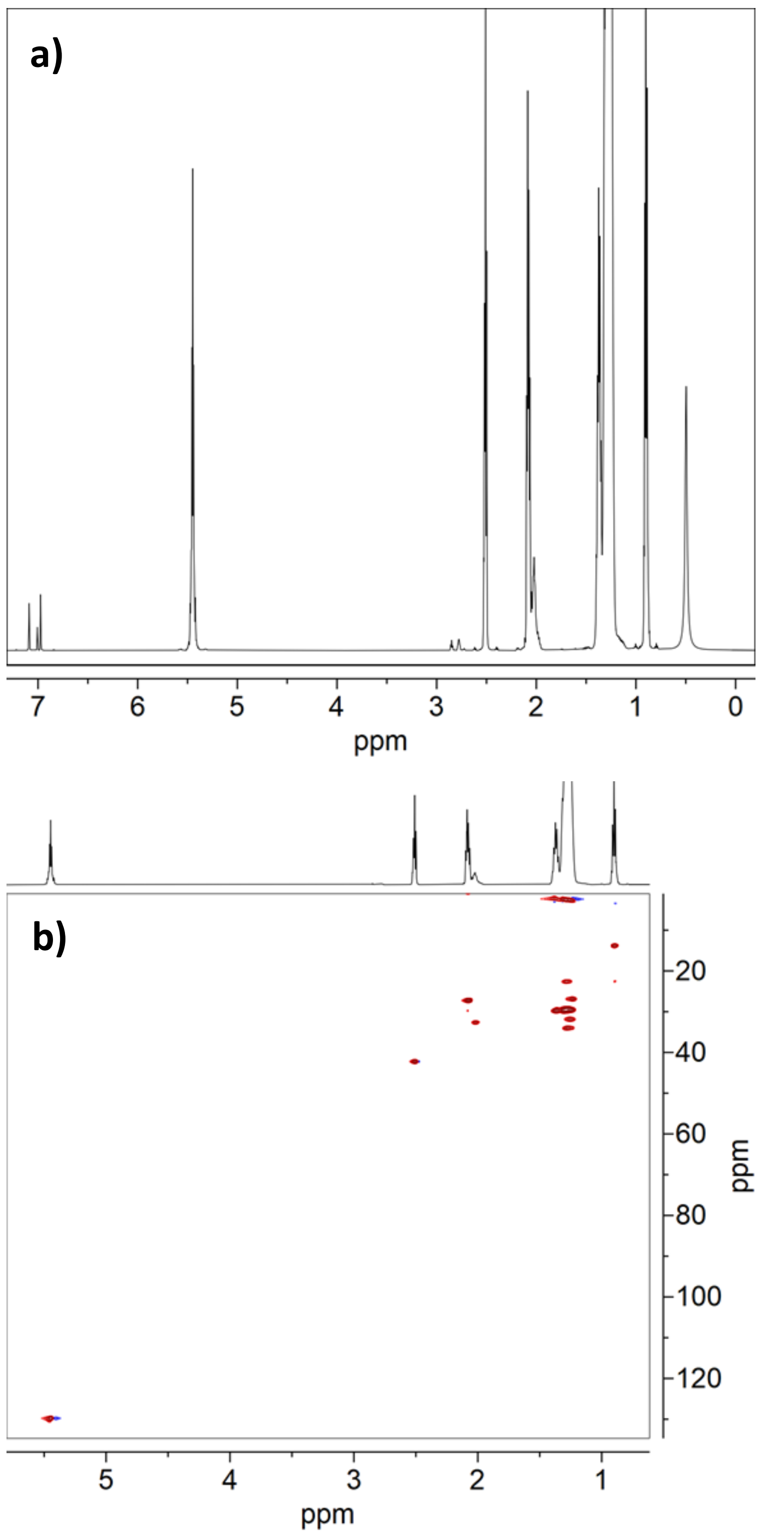

Figure S21. (a) ^{1}H-NMR and (b) ^{1}H-^{13}C HSQC spectra of pristine OA in toluene-d_8 (14.1 T, 298 K).

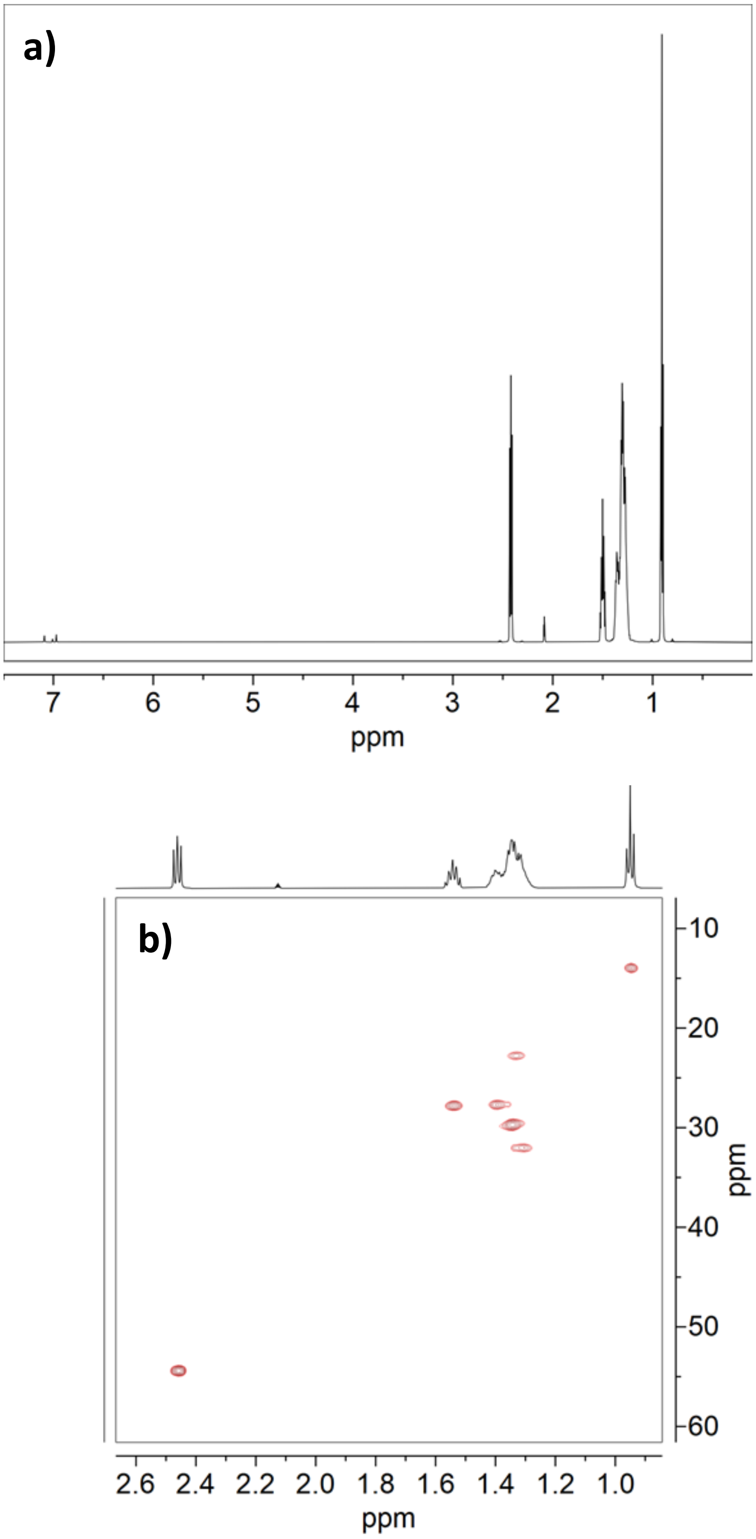

Figure S22. (a) ^{1}H-NMR and (b) ^{1}H-^{13}C HSQC spectra of pristine TOA in toluene-d_8 (14.1 T, 298 K).

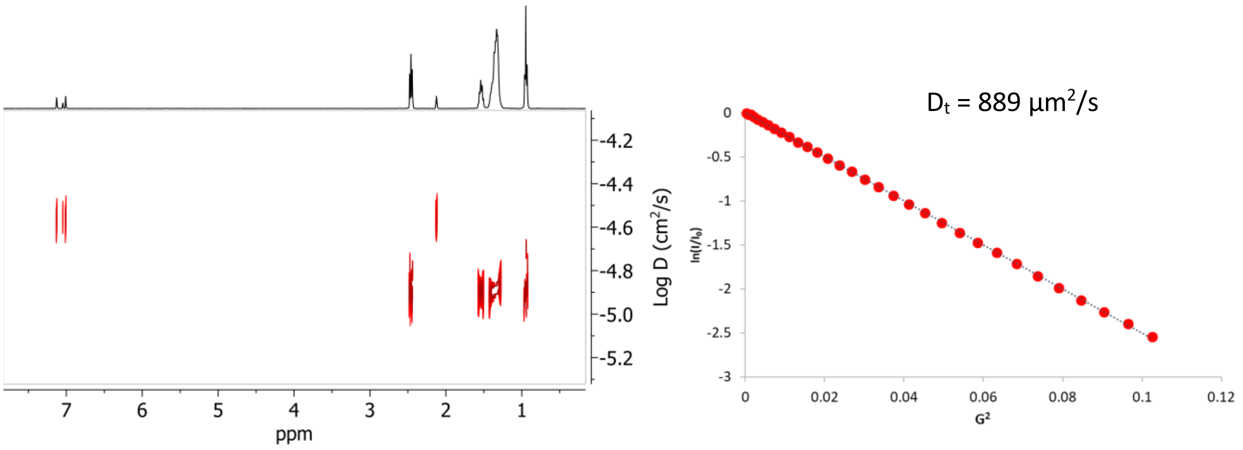

Figure S23. Left: ^{1}H DOSY spectrum of pristine TOA (298 K, 9.4 T, toluene-d_8). Right: Decay of a resonance of TOA as a function of the squared gradient strength, whose slope affords the diffusion coefficient D_t.

References

(1) Zhu, D.; Bellato, F.; Bahmani Jalali, H.; Di Stasio, F.; Prato, M.; Ivanov, Y. P.; Divitini, G.; Infante, I.; De Trizio, L.; Manna, L. $ZnCl_2$ Mediated Synthesis of InAs Nanocrystals with Aminoarsine. *J. Am. Chem. Soc.* **2022**, *144*, 10515-10523.

(2) Ginterseder, M.; Franke, D.; Perkinson, C. F.; Wang, L.; Hansen, E. C.; Bawendi, M. G. Scalable Synthesis of InAs Quantum Dots Mediated through Indium Redox Chemistry. *J. Am. Chem. Soc.* **2020**, *142*, 4088-4092.

(3) Çakan, A.; Sevik, C.; Bulutay, C. Strained Band Edge Characteristics from Hybrid Density Functional Theory and Empirical Pseudopotentials: GaAs, GaSb, InAs and InSb. *J. Phys. D: Appl. Phys.* **2016**, *49*, 085104.

(4) Chao, C. Y.-P.; Chuang, S. L. Spin-Orbit-Coupling Effects on the Valence-Band Structure of Strained Semiconductor Quantum Wells. *Phys. Rev. B* **1992**, *46*, 4110-4122.

(5) Indium Arsenide (InAs), Band Structure, Energies of Symmetry Points. In *Landolt-Börnstein - Group III Condensed Matter. Group IV Elements, IV-IV and III-V Compounds. Part B - Electronic, Transport, Optical and Other Properties.*, Madelung, O., Rössler, U., Schulz, M. Eds.; Vol. 41A1β; Springer Materials

(6) Pryor, C. Eight-Band Calculations of Strained InAs/GaAs Quantum Dots Compared with One-, Four-, and Six-Band Approximations. *Phys. Rev. B* **1998**, *57*, 7190-7195.

(7) Electronic Energy-Band Structure. In *Physical Properties of III-V Semiconductor Compounds*, 1992; pp 75-117.

(8) Vurgaftman, I.; Meyer, J. R.; Ram-Mohan, L. R. Band Parameters for III–V Compound Semiconductors and Their Alloys. *J. Appl. Phys.* **2001**, *89*, 5815-5875.
